# Screening Tests for the Interaction of *Rubus idaeus* and *Rubus occidentalis* Extracts with Antibiotics against Gram-Positive and Gram-Negative Human Pathogens

**DOI:** 10.3390/antibiotics13070653

**Published:** 2024-07-15

**Authors:** Rafał Hałasa, Urszula Mizerska, Marta Kula, Mirosława Krauze-Baranowska

**Affiliations:** 1 Department of Pharmaceutical Microbiology, Medical University of Gdansk, Al. Gen. J. Hallera 107, 80-416 Gdansk, Poland; 2 Department of Polymeric Nanomaterials, Centre of Molecular and Macromolecular Studies, Polish Academy of Sciences, ul. Sienkiewicza 112, 90-363 Lodz, Poland; urszula.mizerska@cbmm.lodz.pl; 3 Moderna Poland sp.zoo, Rondo Ignacego Daszyńskiego 1, 00-843 Warszawa, Poland; marta.kula@modernatx.com; 4 Department of Pharmacognosy, Medical University of Gdansk, Al. Gen. J. Hallera 107, 80-416 Gdansk, Poland; miroslawa.krauze-baranowska@gumed.edu.pl

**Keywords:** additivity, antagonisms, antibiotics, double-disk synergy test, extracts, *Rubus* sp., synergism

## Abstract

WHO (World Health Organization) reports from recent years warn about the growing number of antibiotic-resistant bacterial strains. Therefore, there is an urgent need to constantly search for new substances effective in the fight against microorganisms. Plants are a rich source of chemical compounds with antibacterial properties. These compounds, classified as secondary metabolites, may act independently or support the action of currently used antibiotics. Due to the large number of metabolites isolated from the plant kingdom and new plant species being studied, there is a need to develop new strategies/techniques or modifications of currently applied methods that can be used to select plant extracts or chemical compounds isolated from them that enter into positive, synergistic interactions with currently used antibiotics. One such method is the dual-disk synergy test (DDST). It involves the diffusion of active compounds in the agar environment and influencing the growth of microorganisms grown on it. The method was used to assess the interaction of extracts from the fruit and shoots of some cultivated varieties of *Rubus idaeus* and *Rubus occidentalis* with selected antibiotics. The research was conducted on strains of bacteria pathogenic to humans, including *Staphylococcus aureus*, *Corynebacterium diphtheriae*, *Escherichia coli*, *Pseudomonas aeruginosa*, *Helicobacter pylori*, and *Candida albicans*, showing synergy, antagonism, or lack of interaction of the tested substances—plant extract and antibiotic. As a result, it was found that the diffusion method is useful in screening tests to assess the impact of antibiotic–herbal substance interactions on Gram-positive and Gram-negative microorganisms.

## 1. Introduction

The natural environment has huge plant resources, and they vary depending on the climatic zones. Herbal substances contain multi-component mixtures of various biologically active compounds, such as phenolic compounds, terpenoids, essential oils, alkaloids, lectins, and many others [1,2,3]. Many teams of researchers are looking for new chemical compounds with high biological activity and virtually no side effects. Currently, scientists’ research interests focus mainly on plants, and especially on secondary metabolites with potential anticancer and/or antimicrobial effects [1].

For most compounds isolated from plants, biological properties have been determined, among others, to have antioxidant, anti-inflammatory, and antimicrobial properties. The mechanisms of action of plant metabolites on microorganisms differ from those of currently used antibiotics, which may reduce the risk of cross-resistance and is due to differences in their chemical structures.

Some of them, e.g., phenolic components of essential oils, are known for their strong antimicrobial activity; others, in combination with currently used antibiotics, may become substances that modify bacterial resistance [3,4,5]. In recent years, many studies have focused on herbal extract–antibiotic interactions [3,6], especially since an increase in the antibacterial activity of aminoglycosides, quinolones, macrolides, and tetracyclines in the presence of phytochemicals has been demonstrated [3,7]. Multidrug therapies may have additive or synergistic effects, and sometimes antagonistic effects may be observed. A negligible effect is observed when two substances have the same binding site in the cell. An additive effect occurs when it is the sum of the effects of individual substances against a given microorganism. Synergism means that the strength of activity of a combination of substances is greater than the sum of the effects of individual compounds. However, the antagonistic effect leads to a decrease in the activity of the combination of substances compared to their individual activity [3,8].

It is generally believed that extracts obtained using organic solvents, including ethanol and methanol, have greater antibacterial potential than water extracts. This is due to the dissolution of more active herbal ingredients with antimicrobial properties in organic solvents [9,10].

All currently used methods for determining the susceptibility of microorganisms are standardized, controlled, and updated by the European Committee on Antimicrobial Susceptibility Testing (EUCAST) or the Clinical and Laboratory Standards Institute (CLSI). In vitro methods used in clinical laboratories are divided into phenotypic and genotypic. The most popular phenotypic methods are diffusion and well techniques (qualitative methods) and dilution with agar or broth (quantitative methods). These techniques can be used to pre-select plant extracts with antimicrobial activity [11].

In the disk diffusion test, a bacterial suspension with a density of 1 × 10^8^ CFU/mL (McFarland 0.5) is inoculated uniformly onto the surface of a Müller–Hinton (MH) agar plate [12]. A paper disk (6 mm in diameter) containing a standard amount of antimicrobial substance is placed on the agar surface and allowed to diffuse into the medium. After incubation in the growing bacteria, a clear area of “no growth” appears around the disk—a zone of inhibition. Its size depends on the diffusion rate of the tested substance and the degree of sensitivity of the microorganism. Strains resistant to the antimicrobial compound reach the edge of the disk. The diameter of the inhibition zone is analyzed based on the accepted interpretation standards for various antibiotics. The double-disk synergy test is a test commonly recommended by EUCAST to detect extended-spectrum β-lactamase (ESBL) resistance mechanisms in Gram-negative bacilli [13,14]. It is also used to detect synergisms between different antibiotics in combating resistant bacteria. This test is a variation of the disk diffusion method, in which two separate disks containing two substances are placed at a distance no greater than the limits of the growth inhibition zones of the compounds. After incubation, any deformations and growth inhibition are observed and interpreted according to Section 3.4. Another variation of the disk diffusion method involves adding the concentrated herbal extract to a cooled agar medium and pouring it onto Petri dishes. The bacterial suspension is then inoculated and antibiotic disks are placed. After incubation, zones of inhibition are checked and compared to established interpretation standards for various antibiotics. If the zone of inhibition increases compared to the norm, this is interpreted as synergism/additivity; if the zone decreases, this is interpreted as antagonism. On the other hand, when the growth inhibition zone remains unchanged, is referred to as no impact [15,16].

In well diffusion agar, the bacterial inoculum is mixed with a liquid, cooled agar medium; poured onto a Petri dish; and allowed to solidify. Then, using a sterile drill (4–8 mm in diameter), wells are cut and the test substances are introduced into each well. After incubation, the plate is observed for obvious zones of inhibition around the well. A variation of this method involves evenly spreading the inoculum suspension on a solid agar plate and adding an antimicrobial substance to each well [2,15,16].

Other methods useful for testing the antibacterial activity of herbal extracts are bioautography (based on planar chromatography techniques), the broth and agar dilution methods, and the killing time assay and the checkerboard assay. All quantitative methods are precise and enable the determination of active concentrations such as MIC—minimum inhibitory concentration and MBC—minimum bactericidal concentration. However, they are of little use in screening tests due to the high consumption of reagents and their absorbent properties [2,15,16].

Depending on the extraction method and solvents used, the composition of the obtained extracts may vary in terms of quantity. The most commonly used method for differentiating the antimicrobial activity of the tested herbal extracts is the disk diffusion method. This is a simple technique in which sterile disks soaked in a specific concentration of analyzed extract are placed on bacteria seeded on an agar medium. Samples are most often incubated in an aerobic atmosphere at 37 °C for 24 h. Zones of growth inhibition around the disk indicate the presence of active compounds.

The aim of the study was to assess the usefulness of disk diffusion methods in examining the interactions of herbal extracts with antibiotics in terms of their effect on microorganisms. Moreover, taking into account previous literature data on the antimicrobial activity of extracts from the fruits and shoots of some varieties of *R. idaeus* and *R. occidentalis*, it was also interesting to assess the types of interactions between these extracts and antibiotics in terms of their impact on selected microorganisms. At the same time, based on previously published scientific research results, it was decided to explain the role of some chemical compounds present in the tested extracts in the observed interactions with antibiotics.

## 2. Results

### 2.1. Extract Preparation and Phytochemical Analysis

Methods of preparation and phytochemical analysis of extracts (*R. occidentalis* “Litacz” fruits, *R. idaeus* “Laszka” fruits, *R. idaeus* “Poranna Rosa” fruits, *R. idaeus* “Willamette” shoot) have been described in our recently published article (Hałasa et al.) [17]. The content of phenolic compounds in dry extracts from the fruits and shoots of *R. idaeus* and *R. occidentalis* varieties is presented in Table 1.

### 2.2. Determination of Susceptibility to Antibiotics by Agar Disk Diffusion Method

The zones of bacterial growth inhibition by the antibiotics and extracts used in the tests are included in Table 2 and Table 3.

For diffusion tests, sets of disks recommended by EUCAST and CLSI were used. Different sensitivity to the antibiotics used was observed, depending on the strains. Generally, strains from the ATCC collection appeared to be sensitive to most antibiotics, whereas clinical strains of β-hemolytic *Streptococcus* groups B, *Acinetobacter baumannii* 2021, and *Stenotrophomonas maltophilia* 12755 were predominantly resistant to the antibiotics tested.

### 2.3. Screening for Antibiotic–Extract Interactions Using the Double-Disk Synergy Test (DDST)

**Figure 1:**
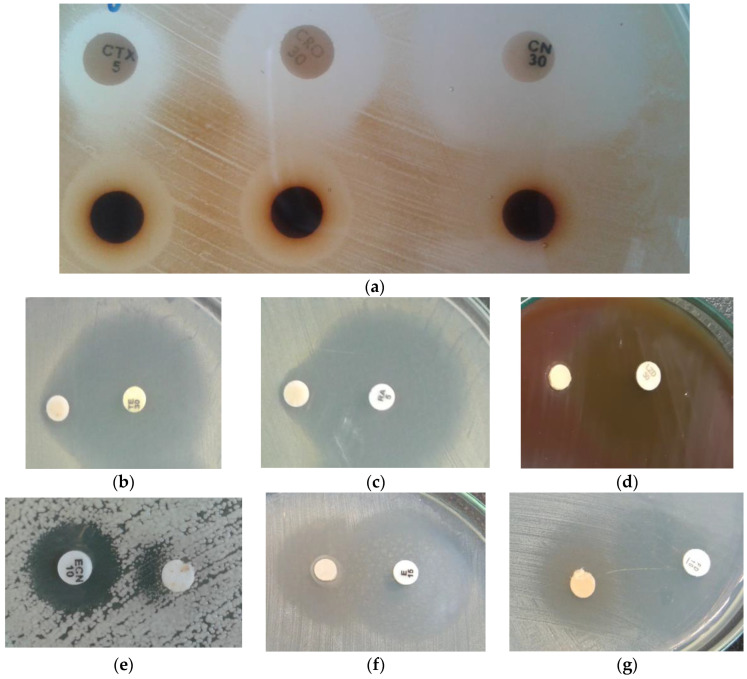

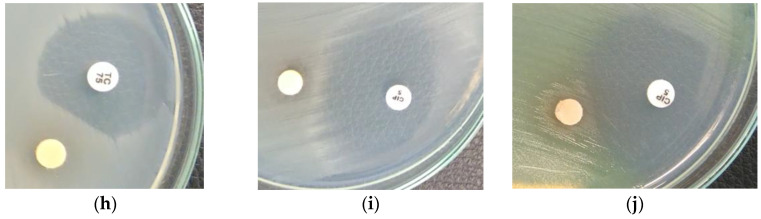
Examples of antibiotic–extract interactions using the double-disk synergy test. (**a**) *S. aureus*—extract of *R. idaeus* “Willamette” shoot—cefazolin (CTX5) and ceftriaxone (CRO30) synergism; extract of *R. idaeus* “Willamette” shoot—gentamicin (GN30)—antagonism; (**b**) *S. aureus*—extract of *R. idaeus* “Poranna Rosa” fruits—tetracycline (Te30)—synergism; (**c**) *S. aureus*—extract of *R. idaeus* “Poranna Rosa” fruits—rifampicin (RA5)—synergism; (**d**) β-hemolytic *Streptococcus* group B—extract of *R. idaeus* “Poranna Rosa” fruits—linezolid (LZD30)—synergism; (**e**) *C. albicans*—extract of *R. idaeus* “Poranna Rosa” fruits—econazole (ECN10)—synergism; (**f**) *S. epidermidis*—extract of *R. idaeus* “Willamette” shoot—erythromycin (E15)—antagonism; (**g**) *S. epidermidis*—extract of *R. idaeus* “Willamette” shoot—fusidic acid (FD10)—antagonism; (**h**) *E.coli* extract of *R. occidentalis* “Litacz” fruits—ticarcillin (TC75)—antagonism; (**i**) *P. vulgaris—*extract of *R. idaeus* “Poranna Rosa” fruits—ciprofloxacin (CIP5); (**j**) *P. aeruginosa*—extract of *R. idaeus* “Willamette” shoot—ciprofloxacin (CIP5).

Examples of antibiotic–extract interaction using the double-disk synergy test are shown in Figure 1. The results of the DDST test for individual bacteria are shown in the subsequent figures in this chapter.

**Figure 2:**
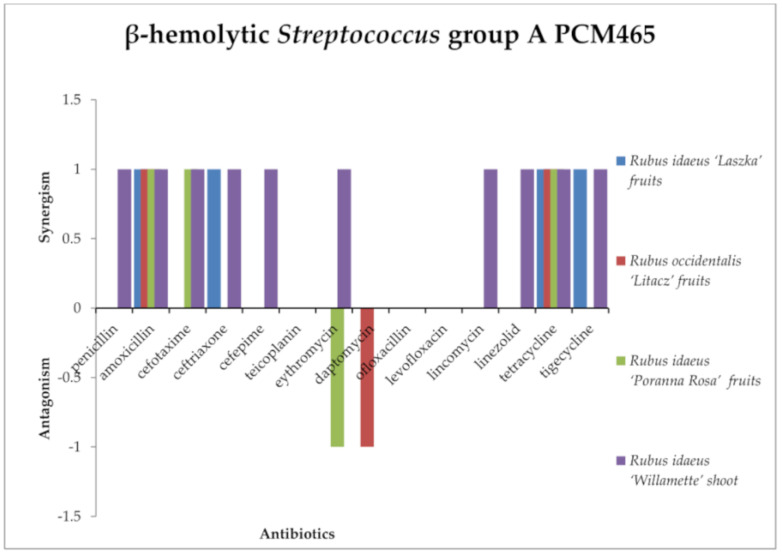
Interactions of *R. idaeus* and *R. occidentalis* extracts with antibiotics against β-hemolytic *Streptococcus* group A PCM465.

**Figure 3:**
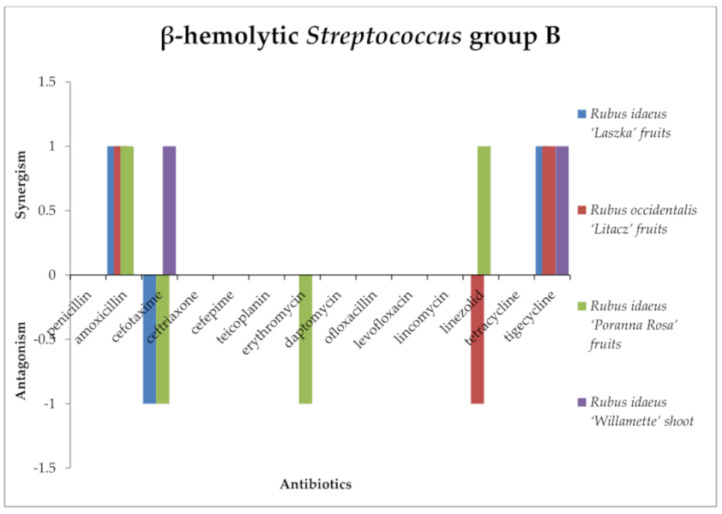
Interactions of *R. idaeus* and *R. occidentalis* extracts with antibiotics against β-hemolytic *Streptococcus* group B.

Among streptococci (Figure 2 and Figure 3), synergy of activity between the tested extracts and antibiotics was observed only against β-hemolytic *Streptococcus*. *R. idaeus* “Willamette” shoot extract showed a synergistic effect with penicillin, amoxicillin, cefotaxime, ceftriaxone, and cefepime (antibiotics from the β-lactam group), as well as erythromycin, lincomycin, linezolid, tetracycline, and tigecycline against β-hemolytic *Streptococcus* group A, with cefotaxime and tigecycline against β-hemolytic *Streptococcus* group B. *R. idaeus* “Laszka” fruit extract presented a synergistic effect with amoxicillin, ceftriaxone, tetracycline, and tigecycline against β-hemolytic *Streptococcus* group A, and amoxicillin and tigecycline against β-hemolytic *Streptococcus* of group B. *R. occidentalis* “Litacz” fruit extract showed a synergistic effect with amoxicillin and tetracycline against β-hemolytic *Streptococcus* group A. *R. idaeus* “Laszka” fruit extract and *R. occidentalis* “Litacz” fruit extract presented synergism with amoxicillin and tigecycline against β-hemolytic *Streptococcus* group B. *R. idaeus* “Poranna Rosa” fruit extract presented a synergistic effect with amoxicillin, cefotaxime, and tetracycline against β-hemolytic *Streptococcus* group A, and against β-hemolytic *Streptococcus* group B with amoxicillin, linezolid, and tigecycline. 

The following compounds were antagonistic to some antibiotics against β-hemolytic *Streptococcus* group A. Only *R. idaeus* “Laszka” fruit extract and *R. idaeus* “Poranna Rosa” fruit extract in combination with erythromycin and daptomycin, respectively, produced an antagonistic effect, whereas, against *Streptococcus* β-hemolytic group B, the antagonistic effect occurred in the case of *R. idaeus* “Laszka” fruit extract (in combination with cefotaxime), *R. idaeus* “Poranna Rosa” fruit extract (in combination with cefotaxime and erythromycin), and Rubus occidentalis “Litacz” fruit extract (in combination with linezolid). 

Against β-hemolytic *Streptococcus* group A PCM465, no interaction was observed between *Rubus idaeus* “Laszka” fruit, *Rubus occidentalis* “Litacz” fruit, *Rubus idaeus* “Poranna Rosa” fruit, and *Rubus idaeus* “Willamette” shoot extracts and teicoplanin, ofloxacillin, or levofloxation. No interaction was observed between *Rubus idaeus* “Laszka” fruit, *Rubus occidentalis* “Litacz” fruit, and *Rubus idaeus* “Poranna Rosa” fruit extracts and penicillin, cefotaxime, cefepime lincomycin, or linezolid. No interaction was observed between *Rubus idaeus* “Laszka” fruit, *Rubus idaeus* “Poranna Rosa” fruit, and *Rubus idaeus* “Willamette” shoot extracts and daptomycin. No interaction was observed between *Rubus idaeus* “Laszka” fruit and *Rubus occidentalis* “Litacz” fruit extracts and erythromycin. No interaction was observed between *Rubus occidentalis* “Litacz” fruit and *Rubus idaeus* “Poranna Rosa” fruit extracts and tigecycline or between *Rubus occidentalis* “Litacz” fruit and *Rubus idaeus* “Poranna Rosa” fruit extracts and ceftriaxone. In studies with β-hemolytic *Streptococcus* group B, no interaction was observed between *Rubus idaeus* “Laszka” fruit, *Rubus occidentalis* “Litacz” fruit, *Rubus idaeus* “Poranna Rosa” fruit, and *Rubus idaeus* “Willamette” shoot extracts and penicillin, ceftriaxone, cefepime teicoplanin, erythromycin, daptomycin, ofloxacillin, levofloxation, lincomycin, linezolid, or tetracycline, or between *Rubus idaeus* “Poranna Rosa” fruit extract with cefotaxime 

**Figure 4:**
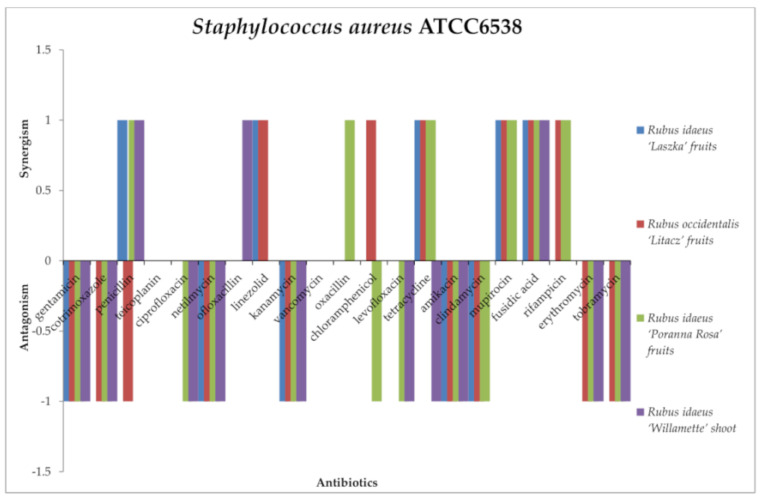
Interactions of *R. idaeus* and *R. occidentalis* extracts with antibiotics against *Staphylococcus aureus* ATCC6538.

**Figure 5:**
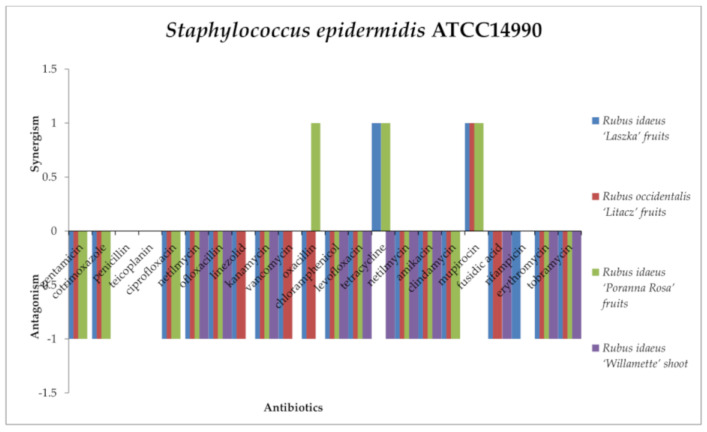
Interactions of *R. idaeus* and *R. occidentalis* extracts with antibiotics against *Staphylococcus epidermidis* ATCC14990.

A synergistic effect of the tested extracts in combination with antibiotics against staphylococci (Figure 4 and Figure 5) was noted. The mixtures of *R. idaeus* “Willamette” shoot extract with penicillin, ofloxacin, and fusidic acid were active against *Staphylococcus aureus* ATCC6538. *R. idaeus* “Laszka” fruit extract was effective with penicillin, linezolid, tetracycline, mupirocin, and fusidic acid against *S. aureus* and with tetracycline and mupirocin against *Staphylococcus epidermidis* ATCC14990. The combination of *R. occidentalis* “Litacz” fruit extract with linezolid, chloramphenicol, tetracycline, mupirocin, fusidic acid, and rifampicin against *S. aureus* yielded the same effect as mupirocin against *S. epidermidis*. In turn, *R. idaeus* “Poranna Rosa” fruit extract in combination with penicillin, oxacillin, tetracycline, mupirocin, fusidic acid, and rifampicin was effective against *S. aureus*. However, the same extract for *S. epidermidis* produced a synergistic effect with oxacillin, tetracycline, and mupirocin.

All extracts in combination with the antibiotics from the aminoglycoside group (gentamycin, kanamycin, amikacin, tobramycin, netilmicin) produced an antagonistic effect against *S. aureus*. Additionally, the same effect was shown by *R. occidentalis* “Litacz” fruit, *R. idaeus* “Poranna Rosa” fruit, and *R. idaeus* “Willamette” shoot extract with co-trimoxazole, erythromycin, and clindamycin. *R. idaeus* “Poranna Rosa” fruit extract and *R. idaeus* “Willamette” shoot extract acted antagonistically with fluoroquinolones (ciprofloxacin, levofloxacin). Antagonism was demonstrated by *R. idaeus* “Poranna Rosa” fruit with chloramphenicol extract, *R. idaeus* “Willamette” shoot extract with tetracycline, R. *idaeus* “Laszka” fruit extract with clindamycin, and *R. occidentalis* “Litacz” fruit extract with penicillin. Against *S. epidermidis*, most of the interactions between the antibiotics used and the extracts turned out to be antagonistic. The exception was penicillin and teicoplanin, where there were no interactions with the extracts. *R. idaeus* “Willamette” shoot extract showed only antagonistic interactions or no interactions with the following antibiotics: gentamycin, co-trimoxazole, penicillin, teicoplanin, ciprofloxacin, mupirocin, vancomycin, oxacillin, mupirocin, and rifampicin. In the case of rifampicin, antagonism was shown only with *R. idaeus* “Laszka” fruit extract.

In the case of *S. aureus* ATCC6538, no interaction was observed between *Rubus idaeus* “Laszka” fruit, *Rubus occidentalis* “Litacz” fruit, *Rubus idaeus* “Poranna Rosa” fruit, and *Rubus idaeus* “Willamette” shoot extracts and teicoplanin, ofloxacillin, or vancomycin. No interaction was observed between *Rubus occidentalis* “Litacz” fruit, *Rubus idaeus* “Laszka” fruit, and *Rubus idaeus* “Willamette” shoot extracts and ciprofloxacin or oxacillin. No interaction was observed between *Rubus idaeus* “Laszka” fruit and *Rubus idaeus* “Laszka” fruit extracts and levofloxacin. No interaction was observed between *Rubus idaeus* “Laszka” fruit and *Rubus idaeus* “Willamette” shoot extracts and chloramphenicol. No interaction was observed between *Rubus idaeus* “Willamette” shoot gentamicin extract and linezolid, clindamycin, mupirocin, rifampicin, azithromycin, or tigecycline. No interaction was observed between *Rubus idaeus* “Poranna Rosa” fruit extract and linezolid or between *Rubus idaeus* “Laszka” fruit and cotrimoxazole. In *S. epidermidis* studies, no interactions were observed in the case of *Rubus idaeus* “Laszka” fruit, Rubus occidentalis “Litacz” fruit, *Rubus idaeus* “Poranna Rosa” fruit and *Rubus idaeus* “Willamette” shoot extracts and penicillin or teicoplanin. No interaction was observed between Rubus occidentalis “Litacz” fruit and *Rubus idaeus* “Willamette” shoot extracts and rifampicin. No interaction was observed between *Rubus idaeus* “Poranna Rosa” fruit and *Rubus idaeus* “Willamette” shoot extracts and linezolid or vancomycin. No interaction was observed between Rubus occidentalis “Litacz” fruit extract with tetracycline or between *Rubus idaeus* “Willamette” shoot extract and gentamicin, cotrimoxazole, ciprofloxacin, oxacillin, clindamycin, mupirocin, ampicillin, or daptomycin.

**Figure 6:**
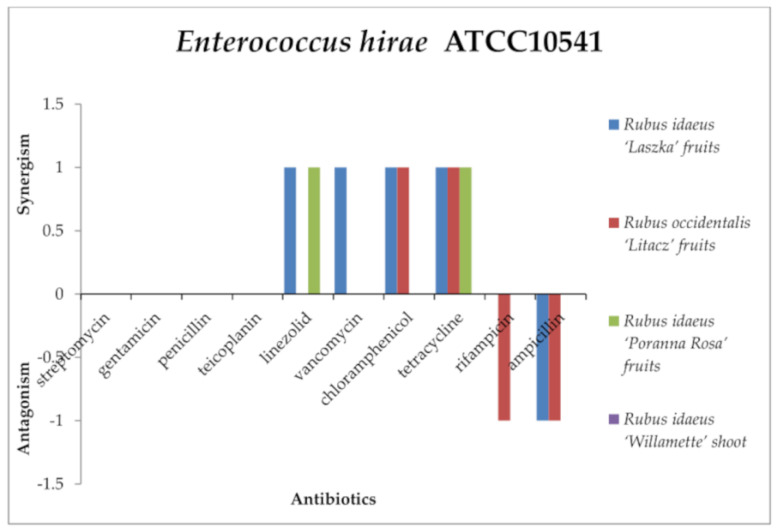
Interactions of *R. idaeus* and *R. occidentalis* extracts with antibiotics against *Enterococcus hirae* ATCC10541.

**Figure 7:**
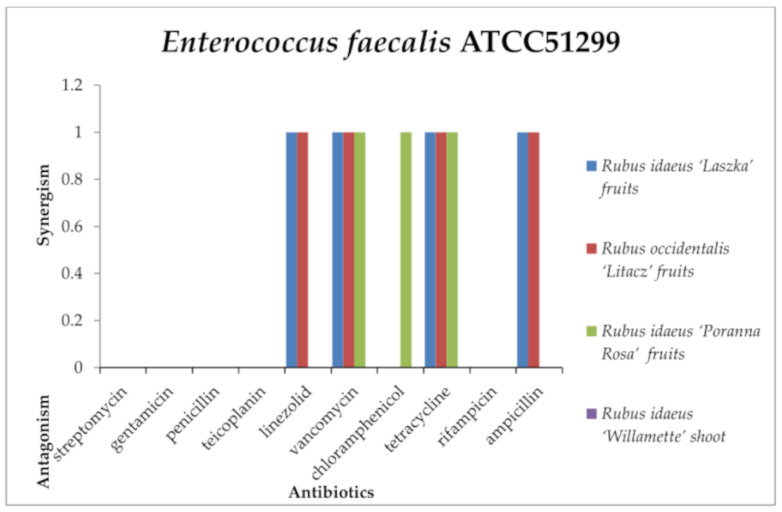
Interactions of *R. idaeus* and *R. occidentalis* extracts with antibiotics against *Enterococcus faecalis* ATCC51299.

The *R. idaeus* “Willamette” shoot extract did not show any effects with the antibiotics used against *Enterococcus hirae* ATCC10541 (Figure 6) and *Enterococcus faecalis* ATCC51299 (Figure 7). Only *R. idaeus* “Laszka” fruit extract with ampicillin and *R. occidentalis* “Litacz” fruit extract with penicillin and rifampicin had an antagonistic effect against *E. hirae*. However, none of the tested extracts showed an antagonistic effect in combination with the antibiotics used against *E. faecalis*.

It was observed that *R. idaeus* “Laszka” fruit extract in combination with linezolid, vancomycin, and tetracycline produced a synergistic effect against *E. hirae* and *E. faecalis*. Additionally, the extract had the same effect with chloramphenicol against *E. hirae* and with ampicillin against *E. faecalis*. The same phenomenon was observed for *R. occidentalis* “Litacz” fruit extract and tetracycline against *E. hirae* and *E. faecalis*; chloramphenicol with *R. occidentalis* “Litacz” fruit extract against *E. hirae*; linezolid, vancomycin, ampicillin against *E. faecalis*; *R. idaeus* “Poranna Rosa” fruit extract with tetracycline against *E. hirae* and *E. faecalis*; *R. idaeus* “Poranna Rosa” fruit extract with linezolid against *E. hirae*; and with vancomycin and chloramphenicol against *E. faecalis*.

In the case of E. hirae, *Rubus idaeus* “Laszka” fruit, *Rubus occidentalis* “Litacz” fruit, and *Rubus idaeus* “Poranna Rosa” fruit extracts did not show any interaction with streptomycin, gentamicin, penicillin, teicoplanin, or vancomycin. Similarly, *Rubus idaeus* “Laszka” fruit and *Rubus idaeus* “Poranna Rosa” fruit extracts did not show any interaction with rifampicin, nor did *Rubus occidentalis* “Litacz” fruit extract with linezolid. *Rubus idaeus* “Poranna Rosa” fruit extract did not show any interaction with chloramphenicol or ampicillin. *Rubus idaeus* “Willamette” shoot extract did not interact with any of the tested antibiotics. In studies, *E. faecalis Rubus idaeus* “Laszka” fruit and *Rubus occidentalis* “Litacz” fruit extracts did not interact with streptomycin, gentamicin, penicillin, teicoplanin, or rifampicin. *Rubus idaeus* “Poranna Rosa” fruit extract did not interact with linezolid or ampicillin. Similarly, *Rubus idaeus* “Laszka” fruit and *Rubus occidentalis* “Litacz” fruit extracts did not interact with chloramphenicol. *Rubus idaeus* “Willamette” shoot extract did not show any interaction with any of the tested antibiotics.

**Figure 8:**
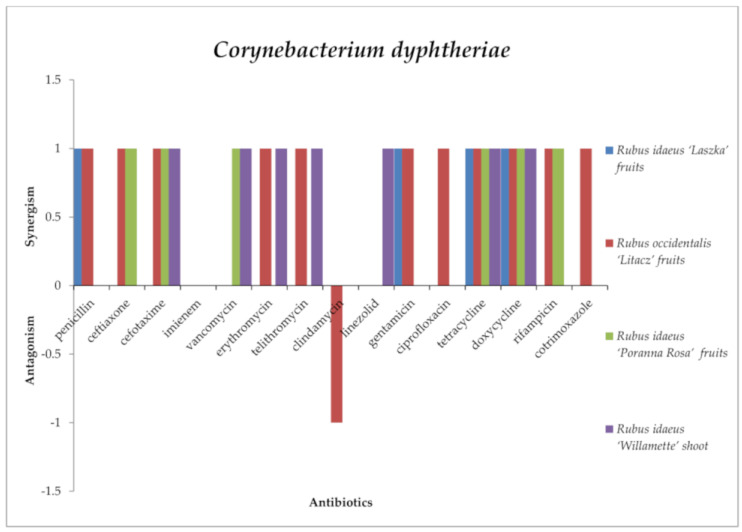
Interactions of *R. idaeus* and *R. occidentalis* extracts with antibiotics against *Corynebacterium diphtheriae*.

Only *R. occidentalis* “Litacz” fruit extract in combination with clindamycin had an antagonistic effect against *Corynebacterium diphtheria* (Figure 8). However, several synergistic interactions between the tested substances were noted. This effect was observed for all the tested extracts with tetracycline and doxycycline. Moreover, the phenomenon of synergism was noticed for the following: *R. idaeus* “Willamette” shoot extract, *R. occidentalis* “Litacz” fruit, *R. idaeus* “Poranna Rosa” fruit with cefotaxime; *R. occidentalis* “Litacz” fruit and *R. idaeus* “Poranna Rosa” fruit extract with ceftriaxone; *R. idaeus* “Willamette” shoot and *R. occidentalis* “Litacz” fruit extract with erythromycin and telithromycin; *R. idaeus* “Laszka” fruit and *R. occidentalis* “Litacz” fruit extract with penicillin and gentamicin; *R. idaeus* “Willamette” shoot; *R. idaeus* “Poranna Rosa” fruit extract with vancomycin; *R. occidentalis* “Litacz” fruit and *R. idaeus* “Poranna Rosa” fruit extract with rifampicin; *R. idaeus* “Poranna Rosa” fruit extract with oxacillin, mupirocin, and fusidic acid; *R. idaeus* “Willamette” shoot extract with linezolid; and *R. occidentalis* “Litacz” fruit extract with cotrimoxazole. 

Against C. diphtheria, *Rubus idaeus* “Laszka” fruit, *Rubus occidentalis* “Litacz” fruit, *Rubus idaeus* “Poranna Rosa” fruit, and *Rubus idaeus* “Willamette” shoot extracts did not show any significant interactions with imipenem. *Rubus idaeus* “Laszka” fruit, *Rubus occidentalis* “Litacz” fruit, and *Rubus idaeus* “Poranna Rosa” fruit extracts did not interact with linezolid. *Rubus idaeus* “Laszka” fruit, *Rubus idaeus* “Poranna Rosa” fruit, and *Rubus idaeus* “Willamette” shoot extracts did not work with clindamycin. *Rubus idaeus* “Laszka” fruit and *Rubus occidentalis* “Litacz” fruit extracts did not interact with vancomycin. Similarly, *Rubus idaeus* “Laszka” fruit and *Rubus idaeus* “Poranna Rosa” fruit extracts did not interact with erythromycin or telithromycin. *Rubus idaeus* “Poranna Rosa” fruit and *Rubus idaeus* “Willamette” shoot extracts did not work with penicillin, gentamicin, ciprofloxacin, or cotrimoxazole. *Rubus idaeus* “Laszka” fruit and *Rubus idaeus* “Willamette” shoot extracts did not interact with cefotaxime, and *Rubus idaeus* “Willamette” shoot extract did not interact with rifampicin.

**Figure 9:**
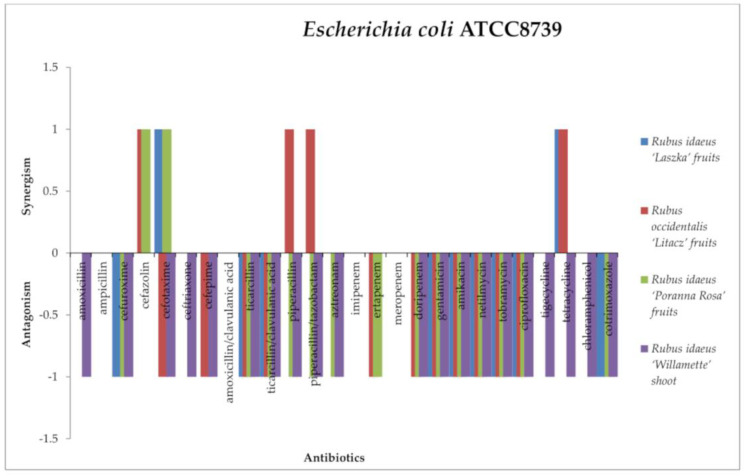
Interactions of *R. idaeus* and *R. occidentalis* extracts with antibiotics against *Escherichia coli* ATCC8739.

In the case of *Escherichia coli* ATCC8739 (Figure 9), the antagonistic effect was exerted by the combination of all extracts with the following antibiotics: ticarcillin, ticarcillin/clavulanic acid, gentamicin, tobramycin, amikacin, and netilmicin (aminoglycoside group), as well as ciprofloxacin and cotrimoxazole. The same effect was observed for the following: *R. occidentalis* “Litacz” fruit, *R. idaeus* “Poranna Rosa” fruit extract, and *R. idaeus* “Willamette” shoot with cefotaxime and doripenem; *R. idaeus* “Poranna Rosa” fruit and *R. idaeus* “Litacz” fruit extract with ertapenem; *R. occidentalis* “Litacz” fruit and *R. idaeus* “Willamette” shoot extract with cefepime; *R. idaeus* “Poranna Rosa” fruit and *R. idaeus* “Willamette” shoot extract with piperacillin, piperacillin/tazobactam, and aztreonam; and *R. idaeus* “Laszka” fruit cefuroxime extract. *R. idaeus* “Willamette” shoot extract showed antagonism with amoxicillin, cefuroxime, ceftriaxone, tetracycline. tigecycline, and chloramphenicol.

A synergistic effect of extracts with antibiotics against *E. coli* was also noted: *R. idaeus* “Laszka” fruit and *R. idaeus* “Poranna Rosa” fruit extract with cefotaxime, *R. idaeus* “Laszka” fruits and *R. occidentalis* “Litacz” fruit extracts with tetracycline, and *R. occidentalis* “Litacz” fruit extract with cefazolin, piperacillin, and piperacillin/tazobactam.

No interactions were demonstrated by *Rubus idaeus* “Laszka” fruits, *Rubus occidentalis* “Litacz” fruits, *Rubus idaeus* “Poranna Rosa” fruits, or *Rubus idaeus* “Willamette” shoot extracts with ampicillin, amoxicillin/clavulanic acid, imipenem, or meropenem. No interactions were demonstrated by *Rubus idaeus* “Laszka” fruits, *Rubus occidentalis* “Litacz” fruits, and *Rubus idaeus* “Poranna Rosa” fruit extracts with amoxicillin, ceftriaxone, tigecycline, or chloramphenicol. No interactions were demonstrated by *Rubus idaeus* “Laszka” fruit and *Rubus idaeus* “Willamette” shoot extracts with cefazolin or ertapenem. No interactions were demonstrated by *Rubus occidentalis* “Litacz” fruit and *Rubus idaeus* “Poranna Rosa” fruit extracts with cotrimoxazole. No interactions were demonstrated by *Rubus idaeus* “Laszka” fruit extract with cefepime, piperacillin, piperacillin/tazobactam, aztreonam, or doripenem. No interactions were demonstrated by *Rubus occidentalis* “Litacz” fruit extract with cefuroxime or aztreonam, and no interactions were demonstrated by *Rubus idaeus* “Poranna Rosa” fruit extract with cefepime and tetracycline.

**Figure 10:**
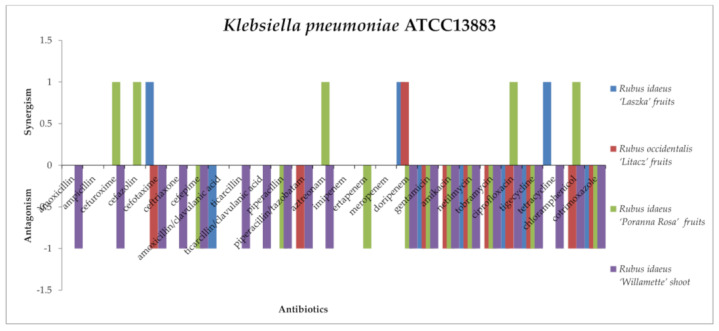
Interactions of *R. idaeus* and *R. occidentalis* extracts with antibiotics against *Klebsiella pneumoniae* ATCC13883.

An antagonistic effect against *Klebsiella pneumoniae* ATCC13883 (Figure 10) was observed for the following combinations: *R. occidentalis* “Litacz” fruit, *R. idaeus* “Laszka” fruit, *R. idaeus* “Poranna Rosa” fruit, and *R. idaeus* “Willamette” shoot extract with gentamicin, amikacin, tobramycin, netilmicin (aminoglycoside group), ciprofloxacin, tigecycline, and cotrimoxazole; *R. occidentalis* “Litacz” fruit and *R. idaeus* “Willamette” shoot extract against cefotaxime, piperacillin/tazobactam, and chloramphenicol; *R. idaeus* “Poranna Rosa” fruit and *R. idaeus* “Willamette” shoot extract with cefepime, piperacillin, and doripenem; *R. idaeus* “Laszka” fruit extract with amoxicillin/clavulanic acid; *R. idaeus* “Poranna Rosa” fruit extract with ertapenem; and *R. idaeus* “Willamette” shoot extract with amoxicillin, cefuroxime, cefotaxime, ticarcillin, ticarcillin/clavulanic acid, aztreonam, and tetracycline.A synergistic effect against *K. pneumoniae* was noted for *R. idaeus* “Laszka” fruit extract with cefotaxime, doripenem, and tetracycline; *R. idaeus* “Poranna Rosa” fruit extract with cefuroxime, cefazolin, aztreonam, ciprofloxacin, and chloramphenicol; and *R. occidentalis* “Litacz” fruit extract with doripenem. 

*Rubus idaeus* “Laszka” fruit, *Rubus occidentalis* “Litacz” fruit, *Rubus idaeus* “Poranna Rosa” fruit, and *Rubus idaeus* “Willamette” shoot extracts with amoxicillin and ampicillin showed no interaction. *Rubus idaeus* “Laszka” fruit, *Rubus occidentalis* “Litacz” fruit, and *Rubus idaeus* “Willamette” shoot extracts with cefuroxime, cefazolin, ticarcillin, and piperacillin showed no interaction. Similarly, *Rubus occidentalis* “Litacz” fruit, *Rubus idaeus* “Poranna Rosa” fruit, and *Rubus idaeus* “Willamette” shoot extracts showed no interaction with amoxicillin/clavulanic. *Rubus idaeus* “Laszka” fruit and *Rubus occidentalis* “Litacz” fruit extracts showed no interaction with ceftriaxone, cefepime, or aztreonam. *Rubus idaeus* “Poranna Rosa” fruit and *Rubus idaeus* “Willamette” shoot extracts showed no interaction with cefotaxime or tetracycline. *Rubus idaeus* “Laszka” fruit, *Rubus occidentalis* “Litacz” fruit, and *Rubus idaeus* “Poranna Rosa” fruit extracts showed no interaction with ticarcillin/clavulanic acid. *Rubus idaeus* “Laszka” fruit, *Rubus idaeus* “Poranna Rosa” fruit, and *Rubus idaeus* “Willamette” shoot extracts showed no interaction with piperacillin/tazobatam, imipenem, or meropenem. *Rubus idaeus* “Laszka” fruit extract showed no interaction with amikacin or chloramphenicol. *Rubus idaeus* “Laszka” fruit extract showed no interaction with ertapenem or tobramycin. *Rubus idaeus* “Willamette” shoot extract showed no interaction with tetracycline.

**Figure 11:**
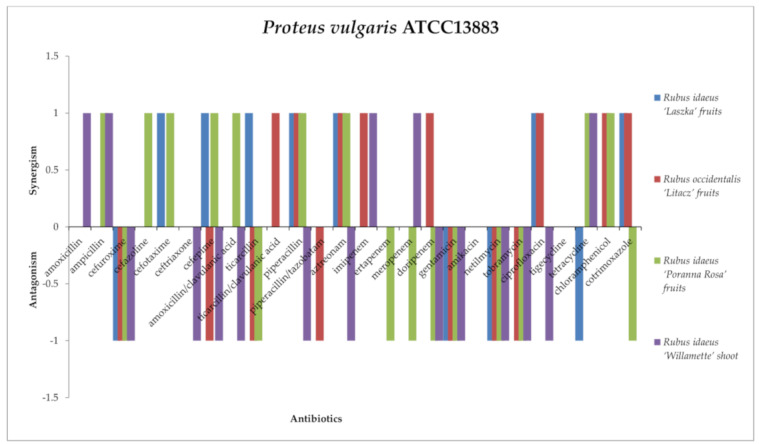
Interactions of *R. idaeus* and *R. occidentalis* extracts with antibiotics against *Proteus vulgaris* NTCT4635.

Antagonistic activity against *Proteus vulgaris* NTCT4635 (Figure 11) was exerted by the following: *R. occidentalis* “Laszka” fruit, *R. idaeus* “Litacz” fruit, *R. idaeus* “Poranna Rosa” fruit, and *R. idaeus* “Willamette” shoot extracts with cefuroxime, gentamicin, netilmicin, amikacin, and tobramycin; *R. occidentalis* “Litacz” fruit and *R. idaeus* “Willamette” shoot extract in the presence of cefepime; *R. idaeus* “Laszka” fruit extract with tetracycline; *R. occidentalis* “Litacz” fruit extract with ticarcillin and piperacillin/tazobactam; *R. idaeus* “Poranna Rosa” fruit extract with ticarcillin, ertapenem, meropenem, doripenem, and cotrimoxazole; and *R. idaeus* “Willamette” shoot extract with ceftriaxone, amoxicillin/clavulanic acid, piperacillin, aztreonam, and ciprofloxacin.

In turn, a synergistic effect of extract with antibiotics against *P. vulgaris* was observed for the following combinations: *R. idaeus* “Willamette” shoot extract with amoxicillin, ampicillin, imipenem, meropenem, and tetracycline; *R. idaeus* “Laszka” fruit extract with cefotaxime, cefepime, ticarcillin, piperacillin, aztreonam, ciprofloxacin, and cotrimoxazole; *R. occidentalis* “Litacz” fruit extract with ticarcillin/clavulanic acid, piperacillin, aztreonam, imipenem, doripenem, ciprofloxacin, chloramphenicol, and cotrimoxazole; and *R. idaeus* “Poranna Rosa” fruit extract with ampicillin, cefazolin, cefotaxime, cefepime, amoxicillin/clavulanic acid, piperacillin, aztreonam, tetracycline, and chloramphenicol.

There was no interaction between *Rubus occidentalis* “Litacz” fruit, *Rubus idaeus* “Poranna Rosa” fruit, *Rubus idaeus* “Laszka” fruit, and *Rubus idaeus* “Willamette” shoot extracts and tigecycline or amikacin; *Rubus idaeus* “Laszka” fruit, *Rubus occidentalis* “Litacz” fruit, *Rubus idaeus* “Poranna Rosa” fruit extracts and amoxicillin or ceftriaxone; *Rubus idaeus* “Laszka” fruit and *Rubus occidentalis* “Litacz” fruit extracts and ampicillin and meropenem; *Rubus idaeus* “Laszka” fruit, *Rubus occidentalis* “Litacz” fruit, and *Rubus idaeus* “Willamette” shoot extracts and cefazolin, amoxicillin/clavulanic acid, or ertapenem; *Rubus idaeus* “Laszka” fruit, *Rubus idaeus* “Poranna Rosa” fruit, and *Rubus idaeus* “Willamette” shoot extracts and ticarcillin/clavulanic acid or piperacillin/tazobatam; *Rubus occidentalis* “Litacz” fruit and *Rubus idaeus* “Willamette” shoot extracts and cefotaxime; *Rubus idaeus* “Laszka” fruit and *Rubus idaeus* “Poranna Rosa” fruit extracts and imipenem; *Rubus idaeus* “Laszka” fruit and *Rubus idaeus* “Willamette” shoot extracts and chloramphenicol; *Rubus idaeus* “Laszka” fruit extract with doripenem or tobramycin; *Rubus occidentalis* “Litacz” fruit extract and tetracycline; *Rubus idaeus* “Poranna Rosa” fruit extract and ciprofloxacin; or *Rubus idaeus* “Willamette” shoot extract with ticarcillin or cotrimoxazole.

**Figure 12:**
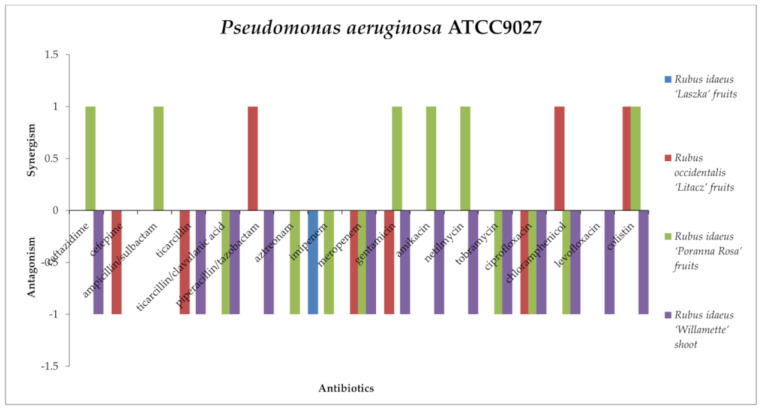
Interactions of *R. idaeus* and *R. occidentalis* extracts with antibiotics against *Pseudomonas aeruginosa* ATCC9027.

The antagonistic effects against *Pseudomonas aeruginosa* ATCC9027 (Figure 12) were exerted by the following: *R. idaeus* “Laszka” fruit extract with imipenem; *R. occidentalis* “Litacz” fruit extract with meropenem; *R. idaeus* “Poranna Rosa” fruit extract with imipenem and meropenem; *R. occidentalis* “Litacz” fruit and *R. idaeus* “Poranna Rosa” fruit extract with ciprofloxacin; *R. idaeus* “Willamette” shoot extract with ciprofloxacin and levofloxacin; *R. occidentalis* “Litacz” fruit extract with gentamicin; *R. idaeus* “Poranna Rosa” fruit extract with tobramycin; *R. idaeus* “Willamette” shoot extract with gentamicin, netilmicin, and tobramycin; *R. occidentalis* “Litacz” fruit extract with cefepime and ticarcillin; *R. idaeus* “Poranna Rosa” fruit extract with ticarcillin/clavulanic acid and aztreonam; *R. idaeus* “Willamette” shoot extract with cefazolin, ticarcillin, ticarcillin/clavulanic acid, piperacillin/tazobactam, and aztreonam; *R. idaeus* “Poranna Rosa” fruit extract with chloramphenicol; and *R. idaeus* “Willamette” shoot extract with chloramphenicol and colistin. 

Synergism against *P. aeruginosa* was demonstrated by *R. occidentalis* “Litacz” fruit extract with piperacillin/tazobactam, chloramphenicol, and colistin, and by *R. idaeus* “Poranna Rosa” fruit extract with ceftazidime, ampicillin/sulbactam, gentamicin amikacin, netilmicin, and colistin. However, *R. idaeus* “Willamette” shoot and *R. idaeus* “Laszka” fruit extract did not show synergistic interactions with the tested antibiotics.

No interactions were observed between *Rubus idaeus* “Laszka” fruit, *Rubus idaeus* “Poranna Rosa” fruit, *Rubus idaeus* “Willamette” shoot extracts and cefepime; *Rubus idaeus* “Laszka” fruit and *Rubus occidentalis* “Litacz” fruit; extracts and ceftazidime, amikacin, netilmicin, tobramycin, chloramphenicol, or ticarcillin/clavulanic acid; *Rubus idaeus* “Laszka” fruit, *Rubus occidentalis* “Litacz” fruit and *Rubus idaeus* “Willamette” shoot extracts and ampicin/sulbactam or aztreonam; *Rubus idaeus* “Laszka” fruits and *Rubus idaeus* “Poranna Rosa” fruit extracts and ticarcillin, piperacillin/tazobactam, or levofloxacin; *Rubus occidentalis* “Litacz” fruit and *Rubus idaeus* “Willamette” shoot extracts and imipenem; *Rubus idaeus* “Laszka” fruit extract and meropenem, gentamicin, ciprofloxacin, or colistin; or *Rubus occidentalis* “Litacz” fruit extract and imipenem.

**Figure 13:**
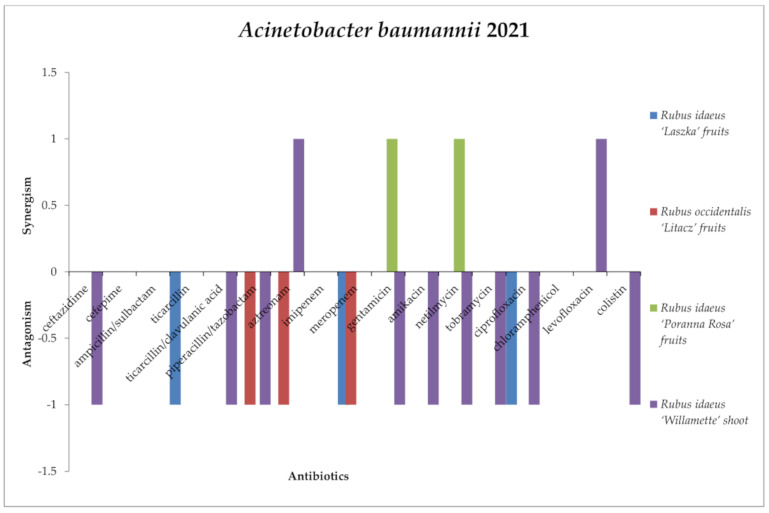
Interactions of *R. idaeus* and *R. occidentalis* extracts with antibiotics against *Acinetobacter baumannii* 2021.

*R. idaeus* “Poranna Rosa” fruit extract did not show antagonism with the antibiotics used against *Acinetobacter baumannii* 2021 (Figure 13). However, the antagonistic effects were exerted by *R. idaeus* “Laszka” fruit extract with ticarcillin, meropenem, and ciprofloxacin; *R. occidentalis* “Litacz” fruit extract with piperacillin/clavulanic acid and meropenem; and *R. idaeus* “Willamette” shoot extract with ceftazidime, ticarcillin/clavulanic acid, piperacillin/tazobactam, gentamicin, amikacin, netilmicin, tobramycin, and colistin.

*R. idaeus* “Willamette” shoot extract and *R. idaeus* “Laszka” fruit extract did not show synergism with any antibiotics used against *Acinetobacter baumannii* 2021. However, synergism of extracts with antibiotics was recorded for *R. occidentalis* “Litacz” fruit extract with piperacillin/tazobactam, chloramphenicol, and colistin, and *R. idaeus* “Poranna Rosa” fruit extract and ceftazidime, ampicillin/sulbactam, gentamicin amikacin, netilmycin, and colistin.

No effects were shown by *Rubus idaeus* “Laszka” fruit, *Rubus occidentalis* “Litacz” fruit, *Rubus idaeus* “Poranna Rosa” fruit, and *Rubus idaeus* “Willamette” shoot extracts with cefepime, ampicin/sulbactam, imipenem, or chloramphenicol; *Rubus idaeus* “Laszka” fruit, *Rubus occidentalis* “Litacz” fruit, and *Rubus idaeus* “Poranna Rosa” fruit extracts with ceftazidim or ticarcillin/clavulanic acid; *Rubus occidentalis* “Litacz” fruit, *Rubus idaeus* “Poranna Rosa” fruit, and *Rubus idaeus* “Willamette” shoot extracts and ticarcillin; *Rubus idaeus* “Laszka” fruit and *Rubus idaeus* “Poranna Rosa” fruit extracts with piperacillin/tazobactam; *Rubus idaeus* “Laszka” fruit, *Rubus occidentalis* “Litacz” fruit, and *Rubus idaeus* “Poranna Rosa” fruit extracts from amikacin, tobramycin, levofloxacin, or colistin; *Rubus idaeus* “Laszka” fruit *and Rubus occidentalis* “Litacz” fruit extracts with gentamicin and netilmycin; *Rubus occidentalis* “Litacz” fruit and *Rubus idaeus* “Poranna Rosa” fruit extracts from ciprofloxacin; *Rubus idaeus* “Laszka” fruit and *Rubus idaeus* “Poranna Rosa” fruit extracts with aztreonam; or *Rubus idaeus* “Willamette” shoot and *Rubus idaeus* “Poranna Rosa” fruit extracts with meropenem.

Against *Stenotrophomonas maltophilia* 12755, antagonistic activity was exerted only by *R. idaeus* “Willamette” shoot extract with ceftazidime, levofloxacin, cotrimoxazole, and colistin.

No effects were shown by *Rubus idaeus* “Laszka” fruit, *Rubus occidentalis* “Litacz” fruit, and *Rubus idaeus* “Poranna Rosa” fruit extracts from levofloxacin, cotrimoxazole, colistin, or ceftazidim.

In turn, against *Helicobacter pylori* ATCC 10231, synergistic effects were exerted only by *R. idaeus* “Laszka” fruit extract, *R. occidentalis* “Litacz” fruit extract, and *R. idaeus* “Poranna Rosa” fruit extract with tetracycline.

**Figure 14:**
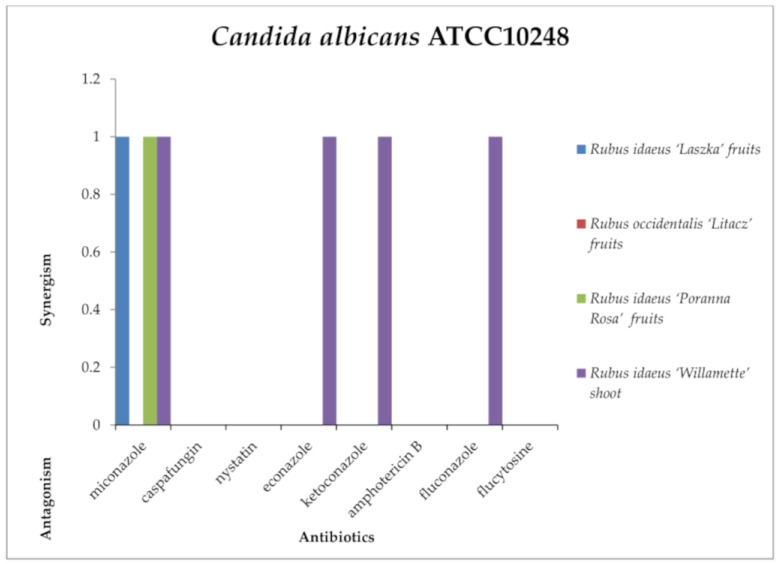
Interactions of *R. idaeus* and *R. occidentalis* extracts with antimycotics against *Candida albicans* ATCC10248.

Against *Candida albicans* ATCC10248 (Figure 14), a synergistic effect was detected with antimycotics *R. idaeus* “Laszka” fruit and *R. idaeus* “Poranna Rosa” fruit extract with miconazole, and with *R. idaeus* “Willamette” shoot extract with miconazole, econazole, ketoconazole, and fluconazole. No effects were shown by *Rubus idaeus* “Laszka” fruit, *Rubus occidentalis* “Litacz” fruit, *Rubus idaeus* “Poranna Rosa” fruit, and *Rubus idaeus* “Willamette” shoot extracts with caspafungin, nystatin, amphotericin B, or flucytosine; *Rubus idaeus* “Laszka” fruit, *Rubus occidentalis* “Litacz” fruit, and *Rubus idaeus* “Poranna Rosa” fruit extracts with econazole and ketoconazole; or *Rubus occidentalis* “Litacz” fruit miconazole extract.

## 3. Materials and Methods

### 3.1. Strains, Growth Medium, Antibiotics

Gram-positive bacteria: β-hemolytic *Streptococcus* group A PCM465, β-hemolytic *Streptococcus* groups B (clinical isolates, collection of the Department of Pharmaceutical Microbiology, Medical University of Gdańsk), *Corynebacterium diphtheria* (collection of the Department of Pharmaceutical Microbiology, Medical University of Gdańsk), *Enterococcus hirae* ATCC10541, *Enterococcus faecalis* ATCC51299, *Staphylococcus aureus* ATCC6538, *Staphylococcus epidermidis* ATCC14990, *Candida albicans* ATCC10231. Gram-negative bacteria: *Escherichia coli* ATCC8739, *Klebsiella pneumoniae* ATCC13883, *Proteus vulgaris* NTCT4635, *Pseudomonas aeruginosa* ATCC9027, *Acinetobacter baumannii* 2021 (collection of the Department of Pharmaceutical Microbiology, Medical University of Gdańsk), *Stenotrophomonas maltophilia* 12755 (clinical isolates, collection of the Department of Pharmaceutical Microbiology, Medical University of Gdańsk), and *Helicobacter pylori* ATCC10231. Columbia agar with 5% sheep blood (Becton Dickinson, Franklin Lakes, NJ, USA) for β-hemolytic *Strpetococcus* groups A and B, (strains growth in 5% CO_2_ atmosphere at 37 °C for 48 h), *Corynebacterium diphtheria*, (growth in an aerobic atmosphere for 48 h at 37 °C). *Enterococcus hirae*, *Enterococcus faecalis, Staphylococcus aureus, Staphylococcus epidermidis*, *Escherichia coli*, *Klebsiella pneumoniae, Proteus vulgaris*, *Pseudomonas aeruginosa*, *Acinetobacter baumanii*, grown in Mueller–Hinton agar (cation-adjusted MH, Becton Dickinson) in an aerobic atmosphere at 37 °C for 48 h. *Helicobacter pylori* grown in tryptic soy broth (TSA, Becton Dickinson) supplemented with 5% sheep blood in microaerophilic conditions (GENbag microaer, BioMerieux, Lyon, France) at 37 °C for 72–96 h (materials from ATCC). *Candida albicans* grown in Sabourauda agar (Biomaxima, Lublin, Poland) at 37 °C for 48 h. 

In our research on antibiotic paper disks (BioMaxima S.A., Lublin, Poland), amikacin 30 µg/mL (AK30), amoxicillin 10 µg/mL (AX10), amoxicillin 25 µg/mL (AX25), amoxacillin/clavulanic acid 3 µg/mL (AMC3), amoxicillin/clavulanic acid 20/10 µg/mL (AMC30), ampicillin 10 µg/mL (AM10), ampicin/sulbactam 20/10 µg/mL (SAM30), azitomycin 15 µg/mL (AZM15), aztreonam 30 µg/mL (ATM30), caspafungi (Kf), cefazolin 5 µg/mL (CTX5), cefepime 30 µg/mL (FEP30), cefotaxime 30 µg/mL (CTX30), cefoxitin 30 µg/mL (FOX30), cefpodoxime 10 µg/mL (CPD10), ceftazidime 30 µg/mL (CAZ30), ceftriaxone 30 µg/mL (CRO30), cefuroxime 30 µg/mL (CXM30), chloramphenicol 30 µg/mL (C30), ciprofloxacin 5 µg/mL (CIP5), clindamycin 2 µg/mL (DA2), colistin 50 µg/mL (CT50), cotrimoxazole 25 µg/mL (SXT25), daptomycin 30 µg/mL (DAP30), doripenem 10 µg/mL (DOR10), doxycycline 30 µg/mL (DO30), econazole 10 µg/mL (ECN10), ertapenem 10 µg/mL (ETP10), erythromycin 15 µg/mL (E15), fusidic acid 10 µg/mL (FA10), gentamicin 10 µg/mL (CN10), gentamicin 120 µg/mL (CN120), imienem 10 µg/mL (IMP10), kanamycin 30 µg/mL (K30), ketoconazole 10 µg/mL (KCA10), levofloxacin 5 µg/mL (LEV5), linezolid 30 µg/mL (LZN30), meropenem 10 µg/mL (MEM10), miconazole 10 µg/mL (MCL10), moxifloxacin10 µg/mL (MUP10), mupirocin 200 µg/mL (MUP200), netilmycin 10 µg/mL (NET10), nystatin 100 units/mL (NS100), ofloxacillin 5 µg/mL (OFX5), optochin (OP), oxacillin 1 µg/mL (OX1), oxacillin 5 µg/mL (OX5), penicillin 10 units/mL (P10), piperacillin 30 µg/mL (PRL30), piperacillin/tazobactam 100/10 µg/mL (TZP110), rifampicna 5 µg/mL (RA5), streptomycin 300 µg/mL (S300), teicoplanin 30 µg/mL (TEC30), telithomycin 15 µg/mL (TELl15), tetracycline 30 µg/mL (Te30), ticarcillin 75 µg/mL (TC75), ticarcillin/clavulanic acid 75 µg/mL (TIM75), tigecycline 15 µg/mL (TGC15), tobramycin 10 µg/mL (Tob10), and vancomycin 5 µg/mL (Va5) were used.

### 3.2. Preparation of Extracts

The methods of preparation and phytochemical analysis (HPLC-DAD-ES-IMS) of extracts (*R. occidentalis* “Litacz” fruit, *R. idaeus* “Laszka” fruit, *R. idaeus* “Poranna Rosa” fruit, *R. idaeus* “Willamette” shoot) have been described in our recently published article (Hałasa et al.) [17].

### 3.3. Determination of Susceptibility to Antibiotics by the Agar Disk Diffusion Method

Disk diffusion for antibiotics was performed according to EUCAST standards. Antibiotic susceptibility was interpreted according to the EUCAST clinical breakpoints (v11.0) (2020). Columbia agar with 5% sheep blood, Muller–Hinton agar, TSA supplemented with 5% sheep blood or Sabourauda agar (Section 3.1), and antibiotic paper disks (Section 3.1) were used. Freeze-dried methanolic extracts were dissolved in sterile deionized water. Sterile filter paper disks for extracts were 6 mm in diameter.

### 3.4. Screening for Extract–Antibiotic Interactions Using a Double-Disk Synergy Test (DDST)

The double–disk synergy test (DDST) is a test commonly recommended by the EUCST to detect extended-spectrum β-lactamase (ESBL) resistance mechanisms in Gram-negative bacilli [13,14]. It is also used to detect synergisms between different antibiotics in combating resistant bacteria. 

**Figure 15:**
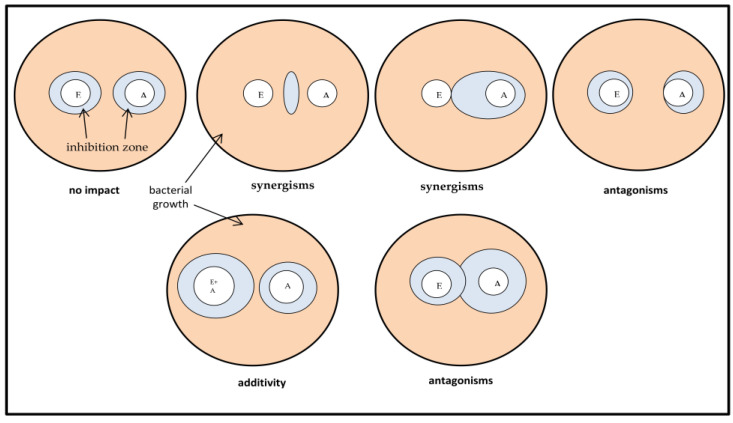
Diagram showing extract (E) –antibiotic (A) interactions against bacteria using the double–disk synergy test (DDST).

The double-disk synergy test (DDST) was performed with some modifications. An appropriate medium was used depending on the species of microorganism being tested, as well as sterile paper disks (6 mm in diameter) for the extracts and commercially available antibiotic disks. The disks were placed on a plate with a medium inoculated with the tested strain of the microorganism. The distance between the arranged disks should be equal to or greater than the sum of the radii of the growth inhibition zones of the individual tested components. After pre-incubation, the plates were subjected to further incubation according to the requirements for a given microorganism. After incubation, the results of the analyzed extract–antibiotic interaction were interpreted on the basis of the obtained growth inhibition zones (Figure 15). In the case of additive or indifferent results, two independent zones of growth inhibition were observed. In the presence of a synergistic interaction, enhancement or bridging was observed at or near the interface of two growth inhibition zones. In the case of antagonism, a cutoff near the junction of two inhibition zones or a separation of the inhibition zones was observed. In the absence of growth inhibition zones (lack of activity) for both components separately, only the presence of both at the same time made it possible to inhibit the growth of microorganisms.

In order to check the additivity of the activity of extracts and antibiotics, a study was carried out in which disks with an antibiotic were placed on bacteria and the extract in a volume of 15 µL/mL was added. After incubation, the size of the growth inhibition zones was checked. The control unit consisted of disks containing only the antibiotic.

Since the zones of inhibition of antibiotics against different bacteria are different, and the interactions of synergism and antagonism change the shape of the zone of growth inhibition (mainly antibiotic), we decided to standardize the results we obtained: synergism and additivity were assigned “+1,” antagonism “−1,” and no interactions “0.”

### 3.5. Statistical Analysis

All experiments were performed at least 3 times. Standard deviation was calculated using Microsoft Excel 2010. Inhibition zone data were analyzed in STATISTICA v. 13.3 using the independent samples t-test. For Gram-positive bacteria, lisnezolid zones were used as a reference, and for C albicans, nystatin zones were used. For Gram-negative bacteria, meropenem growth inhibition zones were used as a reference, and for *S. maltophilia* and *H. pylori*, levofloxacin was used. The *p*-value was considered statistically significant when it was less than 0.05.

## 4. Discussion

The overuse and/or inappropriate use of antibiotics as antimicrobial agents by medical professionals in recent decades, and the widespread use of antibiotics in industry, including animal husbandry and agriculture, has led to the spread of antibiotic resistance among microorganisms. Currently, the most resistant nosocomial pathogens of clinical importance form a group called “ESKAPE.” This group includes species such as *Enterococcus faecium*, *Staphylococcus aureus*, *Klebsiella pneumoniae*, *Acinetobacter baumannii*, *Pseudomonas aeruginosa*, and *Enterobacter* spp., abbreviated as “ESKAPE” [18,19]. The rate of increase in resistance among microorganisms is negatively correlated with the number of effective antibiotics and the discovery of new active compounds. In the face of such data, the WHO has recognized the growing level of antibiotic resistance, accompanied by an increasing number of multidrug-resistant microorganisms, as a global problem, a return to the “pre-antibiotic era,” and a serious threat to public health [20,21]. The Center for Disease Control (CDC) reported that 2 million illnesses in the United States each year are caused solely by antibiotic-resistant microorganisms; 23,000 of them are fatal [22]. Moreover, it is estimated that by 2050, 10 million people may die due to infections caused by antibiotic-resistant bacteria [23], and the costs of treating infections with resistant strains will be in the order of millions per year.

As part of the implementation of new strategies for searching for new compounds with antibacterial activity, plant extracts seem to be a potential source of antimicrobial molecules. Biologically active compounds present in plants are divided into different classes depending on their chemical structure and properties: constituents of essential oils, phenols, alkaloids, saponins, and peptides [24]. Phenols constitute the largest group of secondary plant metabolites. Most phenols present in plants with potential antimicrobial activity are classified into six groups: flavonoids, phenolic acids, tannins, stilbenoids, quinones, and coumarins.

The presence of many chemical compounds in extracts obtained from plants and essential oils raises the possibility that more than one mechanism is responsible for the antimicrobial activity. Therefore, it is believed that the herbal extracts act through several antimicrobial mechanisms at the cellular level, e.g., distribution of the cytoplasmic membrane, disruption and inactivation of the external activities of the membrane of Gram-negative bacteria, shedding lipopolysaccharides, coagulation of the internal content of the cell, and prevention of enzyme production [9,10,25].

The popularity of the disk diffusion method in the initial selection of preparations obtained from plants (herbal extracts) in terms of their antibacterial activity is due to its simplicity, low cost, and repeatability. However, many factors influence obtaining a correct and reliable result, such as the selection of the appropriate medium, its pH, the depth of the agar layer, as well as the incubation conditions, including the humidity of the process, which is partly related to the evaporation of water, which is a constituent of culture medium. The solvent used for the extraction of plant material also affects the diameter of the bacterial inhibition zone achieved and the scope of diffusion of active compounds. The possibility of precipitation of water-insoluble compounds on the disk will also block the process of diffusion of the tested substances in the agar and ultimately affect the growth of the used microorganisms [2,26,27]. The molecular size of the compounds contained in the extract will determine the rate of diffusion between the polymerized agar molecules. As a consequence, there may be results of questionable quality. In the case of polar compounds that diffuse more slowly in the agar, the lack of an inhibition zone does not necessarily mean a lack of antimicrobial activity. Some authors emphasize that the main disadvantage of the disk diffusion test is that it is qualitative rather than quantitative and does not distinguish between bactericidal and bacteriostatic effects. It is believed that disk diffusion can be used to screen low-molecular-weight antimicrobial compounds, while well diffusion is preferred for high-molecular-weight compounds [28].

These negative aspects may also affect the application of disk diffusion to the double-disk synergy test. It should also be remembered that when using the double-disk synergy test to assess interactions, attention should be paid to important details such as the size of the growth inhibition zones of both tested components (herbal extract and antibiotic), which translates into the distance of the disks from each other and the area of joint impact of both components on growing microorganisms. 

Despite certain limitations of disk diffusion, presented above, its use in the double-disk synergy test made it possible to determine the interactions between the tested herbal extracts from fruits and stems of some taxa of the *Rubus* genus and selected antibiotics aimed at enhancing the antimicrobial effect. Moreover, after taking into account some differences and similarities in the chemical composition of the tested plant extracts, it made it possible to clarify the type of certain interactions based on the presence of specific biologically active compounds, primarily in terms of their antimicrobial activity (previously described in the literature). It should also be remembered that when using the double-disk synergy test to assess interactions, attention should be paid to important details such as the size of the growth inhibition zones of both tested components (herbal extract and antibiotic), which translates into the distance of the disks from each other and the area of joint impact of both components on growing microorganisms. 

This research used antibiotics, bacterial strains, and one yeast. Interactions between herbal extract and a given antibiotic were observed in relation to extracts from the fruits of two cultivar varieties of red raspberry, namely, *Rubus idaeus* “Laszka” and “Poranna Rosa,” and the fruit of one cultivar of black raspberry, *Rubus occidentalis* “Litacz,” as well as an extract from the shoots of the “Willamette” variety of *R. idaeus*. The tested extracts were analyzed in terms of chemical composition—compounds from the group of polyphenols and simple phenols—using the HPLC-DAD-ESI-MS method, and their contents were determined [17]. All tested extracts contained high levels of polyphenols comprising the groups of ellagitannins, flavonoids, and phenolic acids, and in two of them anthocyanins were also present (*R. idaeus* “Laszka,” *R. occidentalis* “Litacz”). The extract from shoots of *R. idaeus* “Willamette” was characterized particularly by the highest levels of ellagitannins and free and conjugated ellagic acid (4–6-fold and 2-fold more than fruit extracts, respectively). Ellagitannins are classified as natural antibiotics, and their mechanism of antibacterial action includes, among others, iron chelation, inhibition of cell wall synthesis, disruption of the cell membrane, and inhibition of fatty acid biosynthetic pathways [29].

Anthocyanins were present in only two fruit extracts, and the “Litacz” fruit extract contained four times more anthocyanins. Anthocyanins are also classified as polyphenols with antibacterial activity, but the potency of this action is much weaker compared to tannins. Synergistic interactions with antibiotics were revealed, with 33 for the “Willamette” shoot extract, 31 for the “Laszka” fruit extract, 58 for the “Poranna Rosa” fruit extract (no anthocyanins), and 44 for the fruit of the “Litacz” variety.

Our studies showed that all tested extracts presented synergistic effects most often in combination with tetracycline, tigecycline, penicillin, amoxicillin, and cefotaxime against all tested bacteria. Synergy with tetracyclines has been described for gallic acid as a constituent of herbal extracts. Moreover, studies on the effect of gallic acid on MDR (multidrug-resistant) *E. coli* strains have shown a synergistic effect with ampicillin, oxytetracycline, cefoxitin, and cefotaxime and norfloxacin, ofloxacin, amikacin, and tobramycin [4,30]. However, in the case of *P. aeruginosa*, it influenced the integrity of the cell wall, and a synergistic effect was observed with tetracycline, sulfamethoxazole, and streptomycin [31,32]. Synergy of gallic acid with tetracycline effectively inhibits the in vitro growth of Gram-negative and -positive pathogens [33,34]. *S. aureus* treated with gallic acid lost tolerance to low osmotic pressure. In the case of *E. coli* and *P. aeruginosa* cells, gallic acid caused the outflow of intracellular K^+^ ions, which was related to the permeability of the cytoplasmic membrane [32].

However, in the tested extracts from the genus *Rubus*, gallic acid was identified by the HPLC-DAD-ESI-MS method only in the extract from the shoots of *Rubus idaeus* “Willamette.” The phenols contained in the berries caused a decrease in the permeability of the outer membrane, similarly to EDTA *(ethylenediaminetetraacetic acid)*, by chelating divalent cations, which changed the membrane stabilization by intercalation into the membrane structure [32], as shown in the example of *Salmonella enterica* serovar Typhimurium and *S. enterica* serovar Infantis.

Martinia et al. [35] concluded that polyphenols contained in *R. ulmifolius* extracts act on *H. pylori* ion pumps, i.e., enzymes that regulate the flow of copper and other metal cations through membranes. Additionally, quercetin is a chelator of divalent ions [36]. The mentioned ions (Mg^2+^, Ca^2+^) inactivate tetracyclines [18]. A simultaneous destabilization of the cell membrane was observed to be caused by the synergistic effect of quercetin [33,37], epicatechin [38], ellagic acid derivatives, and caffeic and gallic acid. Literature data indicate that the main mode of action is an increase in cytoplasmic membrane permeability (quercetin) [39,40] intercalated into the cytoplasmic membrane (caffeic acid) [39,41]. Siriwong et al. [42] tested the combination of quercetin and ceftazidime against *Streptococcus pyogenes* and observed, in addition to increased permeability of the cytoplasmic membrane, changes in cell morphology, damage to peptidoglycan and the cell membrane, and decreased levels of nucleic acid. However, increased levels of proteins in bacterial cells were also observed. Epicatechin gallate affected the integrity of the cytoplasmic membrane and inhibited bacterial gene expression [38]. Together with quercetin, it influenced the activity of β-lactamase, reduced the content of fatty acids, and increased the permeability of the cytoplasmic membrane [39,40].

The synergy of phytochemicals with penicillin, amoxicillin, and other beta-lactams has previously been reported. A synergistic effect of quercetin with ampicillin, cefradine, ceftriaxone, imipenem, or methicillin has been demonstrated [36]. The synergy mechanism of quercetin with amoxicillin against MDR *Staphylococcus epidermidis* is as follows: inhibition of peptidoglycan synthesis, activation of β-lactamase, reduction of fatty acid content, and increase in cytoplasmic membrane permeability [39,40]. Epicatechin revealed a synergistic effect against clinical strains of MRSA (methicillin-resistant *Staphylococcus aureus*), with beta-lactam antibiotics: ampicillin, ampicillin/sulbactam, cefazolin, cefepime, and imipenem/cilastatin [37]. (−)-Epigallocatechin gallate influences the staphylococcal virulence factors, has a direct effect on the bacterial cell wall of MRSA and MSSA clinical and standard strains, and shows synergism with oxacillin, ampicillin/sulbactam, penicillin, imipenem, banipenem, meropenem tetracycline, and oxytetracycline [43,44,45,46,47,48].

In our research, the action of epicatechin and quercetin is shown in the interactions of all extracts with ampicillin and amoxicillin against Gram-positive bacteria: *Streptococcus* sp., *Staphylococcus* sp., *Enterococcus* sp., and *C. diphtheriae*. The phenomenon of synergism was observed against Gram-negative bacteria (*E. coli*, *K. pneumoniae*, *P. vulgaris*) for “Poranna Rosa” and “Laszka” fruit extracts in combination with the following antibiotics: cefotaxime, cefepime, cefuroxime, cefazoline, and amoxicillin/clavulanic acid. In the case of *P. aeruginosa* and *A. baumanii*, the “Poranna Rosa” fruit extract produced synergism with ceftazidime, amoxicillin/sulbactam, gentamicin, amikacin, netilmicin, and colistin.

Literature data show that caffeic acid produced synergy with cefotaxime against clinical and reference strains of MRSA and MSSA (methicillin-susceptible *Staphylococcus aureus*) [38,39]. In turn, gallic acid presented the same effect with several beta-lactam antibiotics (ampicillin, cefoxitin, cefotaxime). In the extracts we tested, unfortunately, epicatechin was present only in extracts from “Poranna Rosa” and “Willamete,” whereas caffeic acid and gallic acid were detected only in shoot extract, and the amount of quercetin in all the extracts we tested was relatively small. However, it should be emphasized that the tested extracts may contain other ingredients that act synergistically with beta-lactams.

Caffeic acid intercalated into the cytoplasmic membrane, and the compounds showed synergy with clindamycin and erythromycin against clinical and reference strains of MRSA and MSSA [38,39]. Protocatechuic acid ethyl ester acted synergistically with clindamycin against clinical and standard strains of MRSA and MSSA [49]. The presence of both compounds in the extract we tested was visible in the synergism with erythromycin towards β-hemolytic *Streptococcus* group A and *C. diphtheriae*.

In turn, the synergism of rifampicin with the "Poranna Rosa” and “Litacz” extracts in relation to *S. aureus* and *C. diphtheriae* may indicate the effect of quercetin, which, in combination with rifampicin and ciprofloxacin, acted synergistically against MRSA strains [49,50].

Quercetin and its derivatives (rutin [quercetin-3-O-rhamnoglucoside], quercetin-3-glucoside [isoquercetin], quercetin-3-O-rhamnoside–quercitrin) have several other identified mechanisms of action on bacterial cells, including (i) action on fatty acid synthesis pathway II (FASII type II fatty acid synthase). In *P. aeruginosa*, it inhibits the Fab Z enzyme—dehydratase of the beta-hydroxyacyl-acyl carrier protein (ACP), and in *Mycobacterium* it inhibits FASI (type I FAS fatty acid synthase), which interferes with the synthesis of mycolic acids. In turn, in *H. pylori*, it inhibits 3-hydroxyacyl-ACP dehydrase in FAS II [36,51]. The fluidity and stability of the cell membrane can be affected. (ii) Damage to the cell wall by inhibition of D-alanine-D-alanine ligase, which is responsible for the production of the final peptidoglycan precursor, UDPMurNAc-pentapeptide [36,52]. (iii) Activation of β-lactamase [39,40]. Perhaps these mechanisms are also involved in the revealed synergism with beta-lactam antibiotics.

It should be noted that there are synergisms that we cannot explain and that have not been reported so far. Fruit extracts showed synergism with vancomycin and linezolid against *Enterococcus* spp. and with linezolid, fusidic acid, and mupirocin against *Staphylococcus* spp. However, mupirocin, vancomycin, and fusidic acid in combination with the “Poranna Rosa” extract acted synergistically against *C. diphtheriae*. Based on the results obtained, it should be added that *C. diphtheriae* turned out to be a very sensitive bacterium to the combinations of herbal extracts and antibiotics used. Another bacterium with unusual synergisms in contrast to other Gram-negative bacilli turned out to be *P. vulgaris*. Only against this bacterium did the extract from the shoots of *R. idaeus* “Willamette” show synergism with imipenem, meropenem, amoxicillin, and tetracycline.

Many studies have shown that antibiotics such as beta-lactams, aminoglycosides, and fluoroquinolones, regardless of their specific targets, induce oxidative stress, and thus participate in bacterial killing by antibiotic ROS (reactive oxygen species) [36].

The literature usually contains information about the synergistic interaction of phytochemicals and their mechanisms of action on bacteria, but there is little research on antagonistic interactions.

The fruit and shoot extracts we tested showed antagonism with aminoglycoside antibiotics (gentamicin, tobramycin, amikacin, netilmicin) against *S. aureus*. *E. coli*, *K. pneumoniae*, and *P. vulgaris*. In the case of *P. aeruginosa* and *A. baumannii*, the antagonism phenomenon was dependent on the type of extract. The extract from the shoots of the “Willamette” variety had an antagonistic effect with all tested antibiotics against both bacteria. In, turn, only “Poranna Rosa” fruit extract (with tobramycin) and “Litacz” fruit extract (with gentamicin) showed antagonism towards *P. aeruginosa*. “Litacz” fruit extract had no effect with aminoglycosides against *P. aeruginosa* or *A. baumannii.*

Studies have shown that isoquercetin presents antagonistic effects with aminoglycoside antibiotics, e.g., neomycin, kanamycin, gentamicin, and amikacin against *E. coli* strains. Moreover, neither isoquercetin nor quercetin affected the activity of aminoglycosides against a multi-resistant strain of *S. aureus* [34,53]. Researchers believe that the mechanism of action of quercetin on *E. coli* and *S. aureus* [38] involves damage to the bacterial cell wall and membrane. Moreover, the activity of extracellular alkaline phosphatase and β-galactosidase, as well as the concentration of soluble proteins in *E. coli* and *S. aureus*, increased significantly. On the other hand, while ATP (adenosine triphosphate) synthase activity increased significantly in *S. aureus*, it remained unchanged in *E. coli* [33]. However, there is a different mechanism at work here; it has been shown that quercetin (and its derivatives) and epicatechin [36] inhibit ATP synthase of the F1F0 type, which is a key enzyme in the interconversion of cellular energy [54]. During ATP synthesis, this large protein complex uses the proton gradient and associated membrane potential to synthesize ATP. It can also reverse and hydrolyze ATP, creating a proton gradient. The proton gradient is necessary for the entry of aminoglycosides (gentamicin, amikacin, neomycin, streptomycin, spectinomycin, tobramycin) into the bacterial cell.

The results of phytochemical studies of the tested extracts, presented in Table 1, combined with the mechanisms of action quoted above, suggest that the shoot extracts containing quercetin and epicatechin have an antagonistic effect against the entire group of aminoglycosides, while the “Poranna Rosa” fruit extract (containing quercin and epicatechin in smaller amounts than in the shoot extract) should have lower activity. However, the lowest level of antagonism or its absence should be produced by “Litacz” fruit extracts and “Laszka” fruit extracts (both extracts contained only quercetin). Our results show that antagonism towards aminoglycosides was present in the action of all extracts, regardless of their composition. However, the strength of the antagonistic effect in relation to the quantitative composition of the extracts could be determined using quantitative methods.

Quercetin and its derivative have other mechanisms of action, i.e., inhibition of the activity of bacterial topoisomerase in MRSA (synergism with ciprofloxacin) [4,38,55]. Ohemeng et al. [56] described the inhibition of *E. coli* DNA gyrase by combining with the B subunit [35]. Studies were also conducted in which the effect on gyrase in *Mycobacterium smegmatis* and *M. tuberculosis* was present [38,57].

Topoisomerase is also a target for a group of antibiotics used in therapy, i.e., quinolones (fluoroquinolones), which also have an additional site of attachment in bacterial cells, i.e., DNA gyrase [18]. Quinolones interact with gyrase in Gram-negative bacterial cells and with topoisomerase in Gram-positive bacterial cells. At this point, an antagonistic effect may occur between the ingredients of the extracts, i.e., quercetin and antibiotics. Antagonism between ciprofloxacin and/or levofloxacin and the tested extract from “Wilamette” shoots (against *S. aureus*, *S. epidermidis*, *E. coli*, *K. pneuminiae*, *P. vulgaris*, *P. aeruginosa*) was shown in our studies. A similar profile of plant extract–antibiotic interactions was recorded for fruit extracts from “Poranna Rosa” and “Litacz.” The extract from *R. idaeus* “Laszka” fruit had a synergistic effect only against *E. coli* and *A. baumannii*.

*Rubus idaeus* “Willamette” shoot extract showed antagonism against Gram-negative bacilli mainly with ticarcillin/clavulanic acid, piperacillin/tazobactam, and aminoglycosides. Moreover, the “Willamette” shoot extract with colistin had an antagonistic effect against *P. aeruginosa*, *S. maltophilia*, and *A. baumannii.* The fruit extract of *R. idaeus* “Laszka” showed antagonism with imipenem, as did meropenem against *P. aeruginosa* and *A. baumannii.* The antagonism of *R. idaeus* “Poranna Rosa” fruit extract against *S. aureus* has been demonstrated. The fruit extract of *R. occidentalis* “Litacz” showed antagonism with cefotaxime against *E. coli* and *K. pneumoniae*, and with ticarcillin and piperacillin against *E. coli*, *K. pneumoniae*, *P. vulgaris*, *P. aeruginosa*, and *A. baumannii*. However, “Poranna Rosa” fruit extract presented antagonism with doripenem, ertapenem, meropenem, piperacillin, and ticarcillin against *E. coli*, *K. pneumoniae*, *P. vulgaris*, and *P. aeruginosa*. In turn, the “Litacz” fruit extract showed antagonism with doripenem, imipenem, ertapenem, and meropenem against *E. coli*, *P. aeruginosa*, and *A. baumannii*. The same extract showed antagonism with cefepime against *E. coli*, *P. vulgaris*, and *P. aeruginosa*, and with cefotaxime against *E. coli* and *K. pneumoniae*. Cotrimoxazole was an antagonist of the shoot extract of *R. idaeus* “Willamette” against *S. aureus*, and with *R. idaeus* “Laszka” fruit extract against *E. coli*, *K. pneumoniae*, and *M. catarrhalis.* The phenomenon of antagonism was observed also in the case of *Rubus idaeus* “Poranna Rosa” fruit extract with cotrimoxazole against *S. aureus*, *E. coli*, *K. pneumoniae* and *P. aeruginosa*. The fruit extract of *R. occidentalis* “Litacz” showed antagonism with cotrimoxazole against *S. aureus, E. coli*, and *K. pneumoniae*. Next, the “Litacz” fruit extract showed antagonism with penicillin against *S. aureus* and *E. hirae*, and with clindamycin against *S. aureus* and *Corynebacterium*. It is important to explain the mechanism of this relationship. It is currently unclear which component of the extracts causes the synergistic interactions.

It should be noted that all beta-lactam antibiotics, which include penicillins [18,58], cephalosporins, carbapenems, and monobactams, bind and block specific penicillin-binding proteins (PBPs), thereby inhibiting peptidoglycan synthesis. PBPs exhibit the activity of D,D-transpeptidases and D,D-carboxypeptidases, which are involved in cross-linking-adjacent pentapeptide chains of peptidoglycan [18,59]. Peptidoglycan (PGN) is a component of the bacterial cell wall, and its metabolism plays a major role in determining the structure and shape of bacterial cells, antibiotic resistance, and host–bacteria interactions. Bacteria produce qualitatively and quantitatively different sets of BPB proteins. This results in different activity and range of action of individual beta-lactams. A hypothesis can be formulated that the antagonism of the tested *Rubus* extracts with some beta-lactam antibiotics results from blocking the target site for this antibiotic—the specific BPB protein. At the same time, this may explain the synergism of the tested *Rubus* extracts with another beta-lactam antibiotic that binds to another PBP protein.

It should be added that the antimicrobial effect of phenols depends on their concentration: At low concentrations, phenols inhibit the activity of microbial enzymes, while at high concentrations they induce protein denaturation [60]. The antimicrobial activity of plant metabolites is generally correlated with antioxidant properties, e.g., phenolic acids (caffeic acid); flavonoids, including their glycoside derivatives; some terpenoids; and others [61,62,63].

Diffusion methods are common due to their simplicity, low cost, and speed, and unambiguous results can be achieved at the same time for many test samples. However, like all agar-based methods, they are laborious and time-consuming. The lack of growth inhibition zones does not always mean a lack of activity of the extract or compound contained in it because their degree of diffusion in the solidified agar medium is different and limited by the degree of their polarity, solubility, and particle size [15]. Klančnik et al. [64] found that the broth dilution method is more suitable for assessing the antimicrobial activity of plant extracts than the agar diffusion method. Moreover, their experiments allowed them to conclude that both methods (dilution and diffusion) gave comparable results only in the case of Gram-positive bacteria. Similar results were obtained by us.

To sum up, our goal was to demonstrate the use of the modified double-disk synergy test as a screening method for examining the interaction of antibiotics with herbal extracts on the example of and using extracts from fruit and shoots of certain species and varieties of raspberries rich in phenolic compounds (polyphenols and simple phenols) with confirmed antimicrobial activity in numerous studies. We are aware that, based on diffusion methods, determining the real level of antimicrobial activity of plant extracts may not always be possible, because not all antimicrobial active substances contained in the tested extract dissolved well and penetrated the agar layer. However, the use of disk diffusion in the double-disk synergy test enabled the demonstration of various types of interactions between herbal extracts and antibiotics, the most important of which was revealing the enhancement of the antimicrobial effect of the antibiotic when combined with an herbal extract rich in phenolic compounds.

Moreover, based on literature data and the results of chromatographic analyses regarding the chemical composition of the tested herbal extracts, it was possible to partially explain the mechanism of the observed synergistic effects between a given antibiotic and the tested herbal extract.

We believe that the best and most accurate method for testing the interactions between extracts and antibiotics is the checkerboard dilution method and FICI (Fractional Inhibitory Concentration Index) determination. However, based on our own experience with *H. pylori* (results published in Hałasa et al. [17]), the disk diffusion method allowed for the demonstration of the synergism of the tested extracts with tetracycline (which probably requires the presence of antibacterially active quercetin in the extract). In turn, checkerboard tests allowed for the determination of the exact concentration and showed synergistic interactions of the extracts with doxycycline and levofloxacin, as well as additive interactions with amoxicillin.

We believe that our proposed solution, which involves the use of the disk diffusion method to screen for the determination of the interaction of an herbal extract with an antibiotic against bacteria, is effective, characterized by simplicity of implementation and repeatability. This is confirmed by the interactions between antibiotics and herbal extracts revealed in our research—extracts from fruits of some varieties of *R. idaeus* and *R. occidentalis* and an extract from the shoots of one variety of *R. idaeus*.

## 5. Conclusions

The results of our research show that the proposed method—disk diffusion in the double-disk synergy test (DDST)—allows for the assessment of the interactions between the tested herbal extracts and antibiotics and their impact on the growth of some microorganisms. Using this test, a number of synergistic interactions were revealed between extracts from the fruit and shoots of some cultivars of the *Rubus* genus. *Rubus* extracts, having shown antimicrobial activity in previous studies, also revealed antagonistic effects with some antibiotics. The number of observed antagonistic interactions was greater than the number of observed synergistic interactions. However, extracts from the *Rubus* genus that do not contain anthocyanins but do contain high concentrations of ellagitannins and ellagic acid were characterized by a high number of synergistic interactions and the highest number of antagonistic interactions. Other compounds occurring in lower concentrations in the tested *Rubus* genus extracts may be involved in the mechanisms of the observed interactions, namely, flavonoids, including quercetin and its derivatives and phenolic acids, including caffeic and gallic acid. A full explanation of the mechanism of these interactions requires further research.

## Figures and Tables

**Table 1:** Content of phenolic compounds in dry extracts from the fruits and shoots of *R. idaeus* and *R. occidentalis* varieties [mg/100 g d. w.] in Hałasa et al. [17].

Chemical Compound	Fruits *R. id.* “PR”	Fruits *R. id.* “Lasz”	Fruits *R. occ.* “Lit”	Shoots *R. id* “Will”
Ellagic acid	32.1	24.6	29.6	455.6
Ellagic acid derivatives				
Ellagic acid pentoside	26.8	56.4	23.5	195.1
Ellagic acid methylpentoside ^1^	24.5	22.9	27.9	51.6
Ellagic acid acetylpentoside ^1^	21	22.7	20.6	174.0
Sum of contents	72.3	102.0	72.0	420.7
Ellagitannins				
Sanguiin H-2 ^1^	19.9	20.0	19.7	44.3
Sanguiin H-6 ^1^	140.5	189.0	82.9	339.5
Sanguiin H-10 isomer ^1^	132.8	84.3	116.1	183.6
Lambertianin C ^1^	–	–	–	31.1
Sum of contents	293.2	293.3	218.7	598.5
Anthocyanins				
Cyanidin 3-*O*-glucoside	–	56.4	253.1	–
Cyanidin 3-*O*-rutinoside ^2^	–	14.0	1195.0	–
Cyanidin 3-*O*-sophoroside ^2^	–	122.1	–	–
Cyanidin 3-*O*-(2^G^-glucosylrutinoside) ^2^	–	16.1	–	–
Cyanidin 3-*O*-(2^G^-xylosylrutinoside)^2^	–	–	1312.0 *	–
Cyanidin 3-*O*-sambubioside ^2^				
Pelargonidin 3-*O*-rutinoside ^2^	–	–	37.4	–
Sum of contents	–	208.6	2797.5	–
Others—phenolic acids, flavonoids				
Gallic acid	–	–	–	20.7
Caffeic acid	–	–	–	74.9
Protocatechic acid	–	–	15.4	20.6
Epicatechin	65.5	–	–	1538.2
Quercetin 3-*O*-glucuronide	15.7	10.1	74.4	166.9

Abbreviations: *R. id.* “PR”—*R. idaeus* “Poranna Rosa,” *R. id.* “Lasz”—*R. idaeus* “Laszka,” *R. occ.* “Lit”—*R. occidentalis* “Litacz,” *R. id.* “Will”—*R. idaeus* “Willamette”; ^1^ calculated on ellagic acid; ^2^ calculated on cyanidin 3-*O*-glucoside; * given as a sum due to separation as a single peak.

**Table 2:** Zones of growth inhibition of Gram-positive microorganisms for antibiotics and extracts obtained by the disk diffusion method.

	β-hemolytic *Streptococcus* Group A PCM465	β-hemolytic *Streptococcus* Group B	*Staphylococcus* *aureus* ATCC6538	*Staphylococcus* *epidermidis* ATCC14990	*Enterococcus hirae* ATCC10541	*Enterococcus faecalis* ATCC51299	*Corynebacterium dyphtheriae*	*Cadida albicans ATCC10248*
Oxacillin	N	N	40 ± 0.1	40 ± 0.2	N	N	N	N
Penicillin	58 ± 0.05	12 ± 0.1	26 ± 0.2	10 ± 0.2	6 ± 0.00	6 ± 0.00	30 ± 0.2	N
Amoxicillin	60 ± 0.05	28 ± 0.05	N	N	N	N	N	N
Ampicillin	N	N	N	N	24 ± 0.1	22 ± 0.2	N	N
Cefotaxime	60 ± 0.13	6 ± 0.00	N	N	N	N	48 ± 0.2	N
Ceftriaxone	25 ± 0.21	6 ± 0.00	N	N	N	N	48 ± 0.25	N
Cefepime	20 ± 0.11	6 ± 0.00	N	N	N	N	N	N
Imipenem	N	N	N	N	N	N	6 ± 0.00	N
Netilmycin	N	N	20 ± 0.2	24 ± 0.2	N	N	N	N
Gentamicin (CN10)	N	N	28 ± 0.25	35 ± 0.2	N	N	50 ± 0.2	N
Gentamicin (CN120)	N	N	N	N	15 ± 0.2	12 ± 0.2	N	N
Streptomycin (S300)	N	N	N	N	11 ± 0.1	10 ± 0.1	N	N
Kanamycin	N	N	23 ± 0.22	23 ± 0.2	N	N	N	N
Amikacin	N	N	20 ± 0.1	25 ± 0.52	N	N	N	N
Tobramycin	N	N	23 ± 0.21	25 ± 0.3	N	N	N	N
Erythromycin	20 ± 0.2	15 ± 0.25	32 ± 0.2	22 ± 0.1	N	N	66 ± 0.34	N
Telithromycin	N	N	N	N	N	N	68 ± 0.35	N
Daptomycin	38 ± 0.21	18 ± 0.2	N	N	N	N	N	N
Clindamycin	38 ± 0.22	6 ± 0.00	25 ± 0.2	40 ± 0.23	N	N	50 ± 0.45	N
Linezolid	42 ± 0.1	32 ± 0.2	42 ± 0.1	50 ± 0.2	30 ± 0.2	26 ± 0.26	70 ± 0.55	N
Ofloxacillin	28 ± 0.25	12 ± 034	32 ± 0.21	20 ± 0.25	N	N	N	N
Ciprofloxacin	N	N	40 ± 0.3	45 ± 0.3	N	N	N	N
Levofloxacin	28 ± 0.31	22 ± 0.25	39 ± 0.25	25 ± 0.25	N	N	N	N
Vancomycin	N	N	25 ± 0.2	26 ± 0.31	20 ± 0.36	14 ± 0.15	40	N
Teicoplanin	28 ± 0.1	16 ± 0.2	20 ± 0.2	25 ± 0.1	20 ± 0.2	15 ± 0.2	N	N
Fusidic acid	N	N	25 ± 0.2	40 ± 0.1	N	N	N	N
Rifampicin	N	N	40 ± 0.13	50 ± 0.22	29 ± 0.21	20 ± 0.26	50 ± 0.2	N
Cotrimoxazole	N	N	35 ± 0.2	30 ± 0.22	N	N	29 ± 0.21	N
Mupirocin	N	N	30 ± 0.35	35 ± 0.22	N	N	N	N
Tetracycline	35 ± 0.2	10 ± 0.21	25 ± 0.2	10 ± 0.11	25 ± 0.15	20 ± 0.32	40 ± 0.1	N
Tigecycline	32 ± 0.21	18 ± 0.21	N	N	N	N	N	N
Chloramphenicol	N	N	35 ± 0.1	19 ± 0.25	29 ± 0.2	9 ± 0.11	N	N
Miconazole	N	N	N	N	N	N	N	14 ± 0.1
Caspafungin	N	N	N	N	N	N	N	15 ± 0.1
Nystatin	N	N	N	N	N	N	N	22 ± 0.1
Econazole	N	N	N	N	N	N	N	15 ± 0.1
Extract of *Rubus occidentalis* “Litacz” fruits	9 ± 0.5 *	9 ± 0.5 *	19 ± 0.1 *	23 ± 0.1 *	6 ± 0.1	6 ± 0.1	20 ± 0.1 *	6 ± 0.1
Extract of *Rubus idaeus* “Laszka” fruits	10 ± 0.5 *	9 ± 0.8 *	20 ± 0.1 *	24 ± 0.1 *	6 ± 0.1	6 ± 0.1	19 ± 0.1 *	6 ± 0.1
Extract of *Rubus idaeus*“ Poranna Rosa” fruits	10 ± 0.5 *	11 ± 0.3 *	24 ± 0.1 *	25 ± 0.1 *	6 ± 0.1	6 ± 0.1	22 ± 0.1 *	6 ± 0.1
Extract of *Rubus idaeus* “Willamette” shoot	13 ± 0.1 *	10 ± 0.1 *	23 ± 0.1 *	25 ± 0.1 *	6 ± 0.1	6 ± 0.1	25 ± 0.1 *	6 ± 0.1

N—not tested. The results are presented as mean values ± standard deviation (±SD) from 3 independent experiments. * *p* < 0.05.

**Table 3:** Zones of inhibition of growth of Gram-negative microorganisms for antibiotics and extracts obtained by the disk diffusion method.

	*Escherichia coli* ATCC8739	*Klebsiella pneumoniae* ATCC13883	*Proteus vulgaris* ATCC13883	*Pseudomonas aeruginosa* ATCC9027	*Acinetobacter* *baumanii* 2021	*Stenotrophomonas maltophilia*12755	*Helicobacter pylori* ATCC10231
Amoxicillin	30 ± 0.21	7 ± 0.1	25 ± 0.21	N	N	N	24 ± 0.2
Amoxicillin/clavulanic acid	25 ± 0.12	11 ± 0.2	24 ± 0.12	N	N	N	N
Ampicillin	20 ± 0.2	6 ± 0.00	24	N	N	N	N
Ampicillin/sulbactam	N	N	N	6 ± 0.00	6 ± 0.00	N	N
Cefuroxime	26 ± 0.12	20 ± 0.23	25 ± 0.23	N	N	N	N
Cefotaxime	25 ± 0.2	28 ± 0.22	25 ± 0.1	N	N	N	N
Ceftriaxone	24 ± 0.25	25 ± 0.21	26 ± 0.21	N	N	N	N
Cefazolin	30 ± 0.24	25 ± 0.1	20 ± 0.00	N	N	N	N
Ceftazidime	N	N	N	31 ± 0.23	N	30 ± 0.5	N
Cefepime	40 ± 0.2	20 ± 0.23	21 ± 0.2	20 ± 0.05	16 ± 0.2	N	N
Ticarcillin	25 ± 0.12	12 ± 0.12	28 ± 0.21	25 ± 0.1	16 ± 0.2	N	N
Ticarcillin/clavulanic acid	25 ± 0.2	24 ± 0.2	35 ± 0.2	30 ± 0.21	N	N	N
Piperacillin	35 ± 0.21	28 ± 0.2	28 ± 0.21	N	N	N	N
Piperacillin/tazobactam	32 ± 0.2	29 ± 0.2	35 ± 0.13	37 ± 0.2	30	N	N
Imipenem	25 ± 0.12	7 ± 0.05	19 ± 0.12	30 ± 0.2	6 ± 0.00	N	N
Meropenem	33 ± 0.1	20 ± 0.2	18 ± 0.13	30 ± 0.2	6 ± 0.00	N	N
Ertapenem	33 ± 0.21	17 ± 0.2	18 ± 0.21	N	N	N	N
Doripenem	31 ± 0.22	6 ± 0.00	20 ± 0.21	N	N	N	N
Aztreonam	25 ± 0.2	6 ± 0.00	24 ± 0.1	35 ± 0.2	N	N	N
Clarithromycin	N	N	N	N	N	N	8 ± 0.12
Netilmicin	17 ± 0.25	N	25 ± 0.1	22 ± 0.11	20 ± 0.1	N	N
Gentamicin	19 ± 0.2	N	25 ± 0.23	18 ± 0.12	N	N	N
Amikacin	18 ± 0.1	26 ± 0.2	26 ± 0.21	25 ± 0.2	N	N	N
Tobramycin	19 ± 0.1	10 ± 0.1	29 ± 0.12	N	N	N	N
Ciprofloxacin	35 ± 0.26	N	40 ± 0.35	40 ± 0.35	N	N	N
Levofloxacin	N	N	N	36 ± 0.56	N	35 ± 0.6	30 ± 0.35
Tetracycline	28 ± 0.2	28 ± 0.2	18 ± 0.34	N	N	N	35 ± 0.1
Tigecycline	20 ± 0.1	N	20 ± 0.1	N	N	N	N
Chloramphenicol	31 ± 0.2	30 ± 0.1	28 ± 0.1	18 ± 0.2	15 ± 0.2	N	N
Colistin	N	N	N	17 ± 0.1	14 ± 0.1	18 ± 0.1	N
Metronidazole	N	N	N	N	N	N	6 ± 0.00
Cotrimoxazole	20 ± 0.2	35 ± 0.35	25 ± 0.25	N	N	15 ± 0.25	N
Extract of *Rubus occidentalis* “Litacz” fruits	6 ± 0.1	6 ± 0.1	6 ± 0.1	6 ± 0.1	6 ± 0.1	6 ± 0.1	6 ± 0.1
Extract of *Rubus idaeus* “Laszka” fruits	6 ± 0.1	6 ± 0.1	6 ± 0.1	6 ± 0.1	6 ± 0.1	6 ± 0.1	6 ± 0.1
Extract of *Rubus idaeus* “Poranna Rosa” fruits	6 ± 0.1	6 ± 0.1	6 ± 0.1	6 ± 0.1	6 ± 0.1	6 ± 0.1	6 ± 0.1
Extract of *Rubus idaeus* “Willamette” shoot	6 ± 0.1	6 ± 0.1	6 ± 0.1	6 ± 0.1	6 ± 0.1	6 ± 0.1	6 ± 0.1

N—not tested. The results are presented as mean values ± standard deviation (±SD) from 3 independent experiments.

## Data Availability

Data are contained within the article.
